# Mathematical model of the Tat-Rev regulation of HIV-1 replication in an activated cell predicts the existence of oscillatory dynamics in the synthesis of viral components

**DOI:** 10.1186/1471-2164-15-S12-S1

**Published:** 2014-12-19

**Authors:** Vitaly A Likhoshvai, Tamara M Khlebodarova, Sergei I Bazhan, Irina A Gainova, Valery A Chereshnev, Gennady A Bocharov

**Affiliations:** 1 Novosibirsk State University, Novosibirsk, Russia; 2 Institute of Cytology and Genetics SB RAS, Novosibirsk, Russia; 3 State Research Center of Virology and Biotechnology "Vector", Koltsovo, Russia; 4 Sobolev Institute of Mathematics, Siberian Branch RAS, Novosibirsk, Russia; 5 Institute of Immunology and Physiology, Ural Branch RAS, Ekaterinburg, Russia; 6 Institute of Numerical Mathematics RAS, Moscow, Russia

**Keywords:** HIV-1, mathematical model, replication, feedback regulation, oscillations

## Abstract

**Background:**

The life cycle of human immunodeficiency virus type-1 (HIV-1) makes possible the realization of regulatory strategies that can lead to complex dynamical behavior of the system. We analyze the strategy which is based on two feedback mechanisms, one mediating a positive regulation of the virus replication by Tat protein via the antitermination of the genomic RNAs transcription on TAR (**t**rans**a**ctivation **r**esponsive) element of the proviral DNA and the second mechanism providing a negative regulation of the splicing of the full-length (9 kb) RNAs and incompletely spliced (4 kb) RNAs via their transport from the nucleus to the cytoplasm. Although the existence of these two regulatory feedback loops has been considered in other mathematical models, none of them examined the conditions for the emergence of complex oscillatory patterns in the intracellular dynamics of viral components.

**Results:**

We developed a mechanistic mathematical model for the Tat-Rev mediated regulation of HIV-1 replication, which considers the activation of proviral DNA transcription, the Tat-specific antitermination of transcription on TAR-element, resulting in the synthesis of the full-length 9 kb RNA, the splicing of the 9 kb RNA down to the 4 kb RNA and the 4 kb RNA to 2 kb RNA, the transport of 2 kb mRNAs from the nucleus to the cytoplasm by the intracellular mechanisms, the multiple binding of the Rev protein to RRE (**R**ev **R**esponse **E**lement) sites on 9 kb and 4 kb RNA resulting in their export to the cytoplasm and the synthesis of Tat and Rev proteins in the cytoplasm followed by their transport into the nucleus. The degradation of all viral proteins and RNAs both in the cytoplasm and the nucleus is described. The model parameters values were derived from the published literature data. The model was used to examine the dynamics of the synthesis of the viral proteins Tat and Rev, the mRNAs under the intracellular conditions specific for activated HIV-1 infected macrophages. In addition, we analyzed alternative hypotheses for the re-cycling of the Rev proteins both in the cytoplasm and the nuclear pore complex.

**Conclusions:**

The quantitative mathematical model of the Tat-Rev regulation of HIV-1 replication predicts the existence of oscillatory dynamics which depends on the efficacy of the Tat and TAR interaction as well as on the Rev-mediated transport processes. The biological relevance of the oscillatory regimes for the HIV-1 life cycle is discussed.

## Background

Periodic oscillations are observed in biological systems across multiple levels of their organization, including a single cell level [1-4]. Some researchers view the individual cell as an oscillator [5]. The oscillatory regimes result from the system regulation mechanisms based upon feedback loops [6]. The oscillatory dynamics of genes expression is widely observed in prokaryotic and eukaryotic cells at the molecular-genetic level. The underlying regulation is considered to be a major mechanism for coordinating various intracellular processes. The specific examples include the oscillations in the NF-κB level during the immune response development [7-9], the Hes1 and Hes7 proteins regulating the somite segmentation during the embryonic development of vertebrates [10,11], the p53 protein controlling the cell apoptosis (a programmed cell death) [12], etc. The key role of the negative regulatory feedback loops in the emergence of oscillations has been pointed out in a number of theoretical studies of natural and artificial gene networks [13-17].

The life cycle of human immunodeficiency virus type-1 (HIV-1) makes possible the realization of regulatory strategies that can lead to qualitatively different outcomes. For example, a high level of virus production can lead to the activation of apoptosis resulting in the cell death or to a persistent production of the infectious virus particles over a period of several months [18]. One of the mechanisms enabling high level production of virus particles is the positive regulatory mechanism of the virus replication by Tat protein, with its mRNA being the product of an alternative splicing of HIV-1 genomic RNA that belongs to the 2 kb classes of mRNAs [19]. The production of Tat protein leads to an augmentation of full-length genomic RNA transcription by at least 25- to 100 fold [20-23]. However, for the production of virus particles both the synthesis and the transport from the nucleus to the cytoplasm of the full-length unspliced 9 kb RNA is needed because this full-length RNA represents the viral genome and the mRNA for the Gag and Gag-Pol proteins synthesis. The availability of the full-length viral genomic RNAs and the Gag proteins in the cytoplasm is required for the budding and the final assembly of the virions (for recent review, see [24]). As the eukaryotic cells have no mechanisms for transporting the intron-containing viral RNA out of the nucleus, the transport is provided by the virus via the Rev-mediated mechanism. The Rev protein is synthesized from the completely spliced viral mRNA [25], which is exported from the nucleus to the cytoplasm by endogenous cellular mechanisms [19]. The Rev protein contains the nuclear localization sequence (NLS) or the nuclear export sequence (NES), which control the shuttling of Rev between the nucleus and the cytoplasm [26-28]. Its appearance in the nucleus followed by the interaction with the Rev-responsive element (RRE) leads to the assembly of a high-affinity complex on unspliced- (9 kb) and incompletely spliced (4 kb) viral mRNA [28] and the export of the above classes of the viral mRNAs out of the nucleus. This results in a down-regulation of the generation of the completely spliced mRNAs and therefore, of the overall synthesis of the Rev and Tat proteins [29].

The availability of two regulatory loops, one representing a positive feedback of the activation of HIV-1 transcription mediated by Tat protein and the second one being a negative feedback loop regulating the Tat and Rev mRNA synthesis, sets up the prerequisite for the emergence of oscillatory regimes in the production of virus particles. The mathematical models of the intracellular HIV-1 kinetics [30-34] developed so far consider the above regulatory feedback loops and reproduce some specific experimental data on the kinetics of HIV-1 replication. However, the existence of an oscillatory dynamics in the production of the viral components in an infected cell has not been examined (or discussed) yet.

We develop a mathematical model for the Tat-Rev mediated regulation of HIV-1 replication to examine the dynamics of the accumulation of the Tat and Rev proteins and the viral RNA in an infected macrophage, persistently producing the virus particles. Two specific hypotheses of the re-cycling (nuclear import/export cycle) of HIV-1 Rev protein are considered. The first hypothesis reflects a widely accepted view that the Rev protein is released from the nuclear export complex in the cytoplasm followed by its binding to importin-β (see, e.g. [19]). The second hypothesis is based on the evidence suggesting that the Rev protein returns into the nucleus directly at the nuclear pore complex without the exit to the cytoplasm [35,36].

The mathematical model calibrated using published experimental data on the processes underlying the functioning of the Tat-Rev regulatory loops predicts the existence of oscillatory dynamics which depends on the efficacy of the interaction between the Tat protein and TAR and on the transport kinetics regulated by the Rev protein.

## Model

The mathematical model of the intracellular HIV-1 replication is specified using the biochemical systems formalism [3]. Two elementary types of reactions are considered, the bimolecular and the monomolecular ones. In this section we apply some standard notation to represent the chemical reactions. The following representation is used for a bimolecular reaction:

$A+B\Leftrightarrow C:k_{1},k_{2}$.

In the above notation, *A*, *B *and *C *refer to the concentration of chemical products whereas *k*_1 _and *k*_2 _denote the rate constant of the forward and reverse reactions. According to the Law of Mass Action [3], the following system of ordinary differential equations (ODEs) corresponds to bimolecular reaction.

$\frac{dA\left(t\right)}{dt}=\frac{dB\left(t\right)}{dt}=-\frac{dC\left(t\right)}{dt}=-k_{1}A\left(t\right)B\left(t\right)+k_{2}C\left(t\right)$.

The equations describe the local rates of changes in the concentrations of the reactants *A*,*B*,*C *in a fixed volume. Following the standard paradigm, an instantaneous mixing of the products in the given volume is assumed.

The monomolecular reaction is specified using the following notation:

$$\left[a\right]\cdot A\to \left[b_{1}\right]\cdot B_{1}+...+\left[b_{n}\right]\cdot B_{n}:k.$$

Here *A*, *B_i _*denote the chemical products concentrations, *k *is the reaction rate constant, and *a*={0,1}, *b_i_*≥0 are the stoichiometric coefficients. The monomolecular reaction is mathematically described with the following system of ODEs

$$\frac{dA\left(t\right)}{dt}=-akA\left(t\right),\;\;\frac{dB_{i}\left(t\right)}{dt}=b_{i}kA\left(t\right),i=1,...,n,$$

which determines the local rates of changes in the concentrations of the reactants in a fixed volume. To simplify the notation of the mono-molecular reactions, we will omit the stoichiometric coefficient of 1. The value of *a* = 0 suggests that the reactant *A *is not transformed in the reaction.

### Elementary subsystems of the model

**Figure 1:**
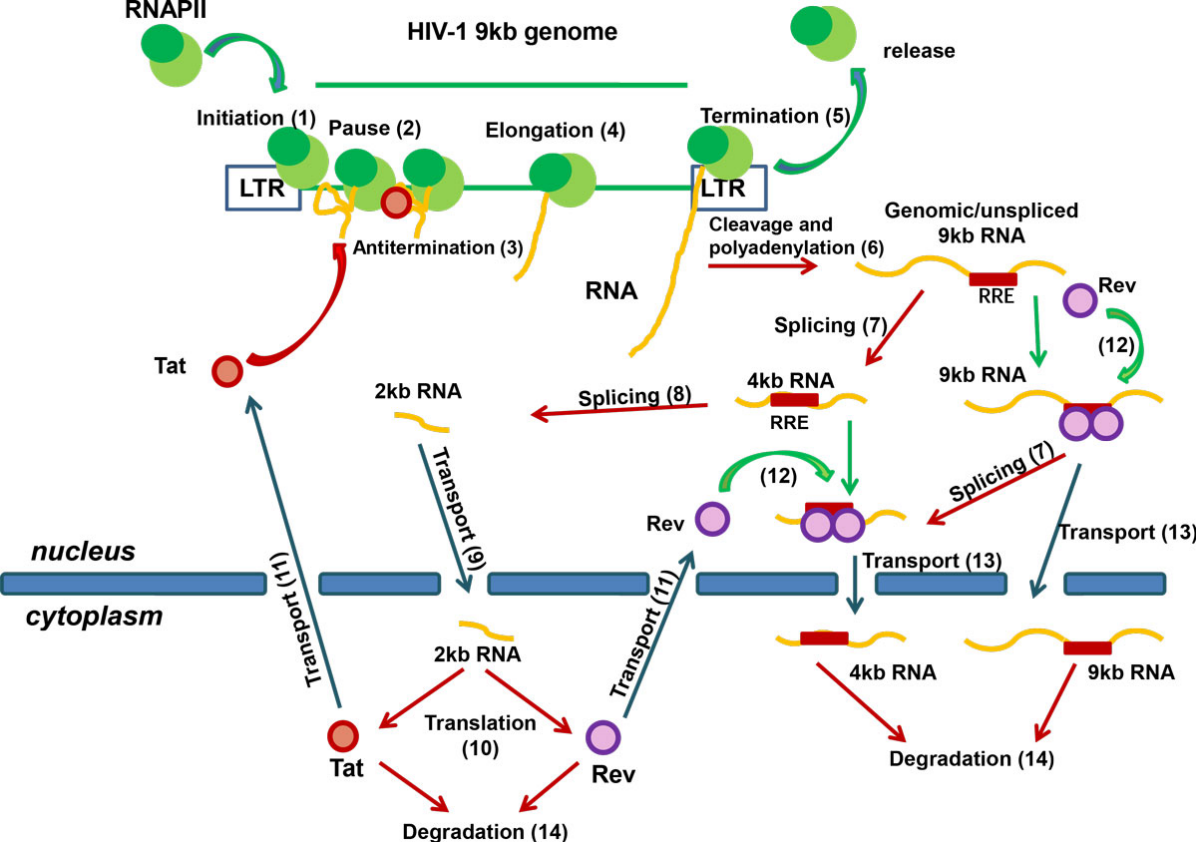
**The scheme of Tat-Rev mediated regulatory circuit of HIV-1 replication used for the model**. The numbering in parentheses corresponds to the numbering of the corresponding subsystem of the biochemical model presented in the section "Elementary subsystems of the model". The description of the subsystems and their respective variables is given in the text.

The biochemical model of the Tat-Rev mediated regulatory network of HIV-1 replication can be decomposed into a set of elementary subsystems. They are specified according to the scheme of the model of HIV-1 replication shown in Figure 1. The specific modules are described below with the corresponding parameters listed in table 1.

**Table 1:** Summary of the model parameters used for numerical simulations of HIV-1 replication.

Subsystem number	Parameter notation	Units	Reference value	Reference
1	$k_{transcr,ini}$	Transcription initiation/(min genome)	0.25 (for non-activated cell)	[64,65]
				
			25 (for activated cell)	
	
	LTR_prom_HIV1	elements/nucleus	1	assigned

2	$k_{delay}$	1/min	1	assigned
	
	*λ*		0.99	estimated

3	$k_{ass,Tat\text{\_}TAR}$	elements/(nucleus min)	0.0017	[45]
		
	$k_{dis,TAT\text{\_}TAR}$	1/min	1	
	
	$k_{antiterm}$	1/min	60	assigned

4	$k_{transcr,elong,i},\;\;\;i=1,...,n_{DNAunit}$	1/min	5.33	derived
	
	$n_{DNAunit}$	dimensionless	20	assigned

5	$k_{transcr,term}$	1/min	60	assigned

6	$k_{modif}$	1/min	60	assigned

7	$k_{splicing,94}$	1/min	0.0415	[38,48,49]
		
8	$k_{splicing,42}$	1/min	0.0415	

	$\delta_{4,Tat}$	dimensionless	0.115	Estimated from [83]
		
	$\delta_{4,Rev}$	dimensionless	0.115	

9	$\begin{array}{c} k_{transp,2kb-mRNA\text{\_}Xxx},\\ Xxx\in \left\{Tat,Rev\right\} \end{array}$	1/min	0.0767	[27]

10	$k_{transl,ini,Xxx},Xxx\in \left\{Tat,Rev\right\}$	1/min	10	assigned
	
	$k_{transl,elong,i},Xxx\in \left\{Tat,Rev\right\}$	1/min	18	derived
	
	$k_{transl,term,n_{Xxx}+1},Xxx\in \left\{Tat,Rev\right\}$	1/min	60	assigned
	
	$n_{Xxx},Xxx\in \left\{Tat,Rev\right\}$	dimensionless	20	assigned

11	$k_{transp,prot\text{\_}Xxx},Xxx\in \left\{Tat,Rev\right\}$	1/min	0.347	[54]

12	$\begin{array}{c} k_{ass,Rev\text{\_}\left(i\right)\text{\_}X\text{\_}nuc},\\ i=1,...,n_{Rev},X\in \left\{9,4\right\} \end{array}$			
		copy/(nucleus min)	0.59	
	$\begin{array}{c} k_{ass,Rev\text{\_}\left(i\right)\text{\_}X\text{\_}cyt},\\ i=1,...,n_{Rev},X\in \left\{9,4\right\} \end{array}$			
				[28]
	$\begin{array}{c} k_{dis,Rev\text{\_}\left(i\right)\text{\_}X\text{\_}nuc},\\ i=1,...,n_{Rev},X\in \left\{9,4\right\} \end{array}$	1/min	8.4	
	
	$\begin{array}{c} k_{dis,Rev\text{\_}\left(i\right)\text{\_}X\text{\_}cyt},\\ i=1,...,n_{Rev},X\in \left\{9,4\right\} \end{array}$			

13	$k_{transp,X,1},X\in \left\{9,4\right\}$	1/min	0	[82]
	
	$\begin{array}{c} k_{transp,X,i},\\ \;i=2,...,n_{Rev,X},X\in \left\{9,4\right\} \end{array}$	1/min	0.0767	[27]
	
	$\gamma_{i},\;\;\;i=1,...,n_{Rev,X}$	dimensionless	12	Quantified following the recycling in the nuclear pore hypothesis

12,13	$n_{Rev,X},X\in \left\{9,4\right\}$	dimensionless	12	[28,52,53]

14	$k_{degr,Xkb-mRNA\text{\_}yyy},X\in \left\{9,4\right\}$, yyy∈{nuc,cyt}			
				
	$\begin{array}{c} k_{degr,2kb\text{\_}mRNA\text{\_}Tat\text{\_}yyy},\\ yyy\in \left\{nuc,cyt\right\} \end{array}$	1/min	0.0029	[58]
				
	$\begin{array}{c} k_{degr,2kb\text{\_}mRNA\text{\_}\text{Re}\;v\text{\_}yyy},\\ yyy\in \left\{nuc,cyt\right\} \end{array}$			
	
	$\begin{array}{c} k_{degr,prot-Xxx\text{\_}nuc},\\ Xxx\in \left\{Tat,Rev\right\} \end{array}$	1/min	0.000722	
				[60]
	$k_{degr,prot-Xxx\text{\_}cyt},Xxx\in \left\{Tat,Rev\right\}$	1/min	0.00289	

Auxiliary parameters	$r_{transcr,elong}$	nts/min	2400	[38,40]
	
	$r_{transcl,elong}$	nts/min	1800	[55,56]
	
	*Nucl_9 kb-RNA*	nucl	9000	[25]
		
	*Nucl_2 kb-mRNA*	nucl	2000	

1. The initiation of transcription from the HIV-1 proviral LTR-promoter <*LTR_prom_HIV*1> leading to the formation of the elongation complex <*TAR_RNApol - II elong*> that is prone to termination at TAR element is represented by:

$$\left[0\right]\cdot LTR\text{\_}prom\text{\_}HIV1\to TAR\text{\_}RNApol-II\text{\_}elong:k_{transcr,ini}\times proV.$$

Here, *k_transcr, ini _*denotes the rate constant of the transcription initiation and *proV *is the number of the proviral DNA genomes in the cell.

2. The passage of the TAR-element by RNA polymerase II. It is assumed that with a probability λ the RNA polymerase II is terminated leading to the formation of short RNA <*microRNA*>, whereas the value (1-λ) gives the probability of the formation of the elongation complex <*DNAunit_RNApol-II_elong_*(1)>, that escaped the termination at the TAR element:

$$TAR\text{\_}RNApol-II\text{\_}elong\to \left[1-\lambda \right]\cdot DNAunit\text{\_}RNApol-II\text{\_}elong\text{\_}\left(1\right)+\left[\lambda \right]\cdot microRNA:k_{delay}.$$

Here, the parameter *k_delay _*is the constant rate of RNA-ploymerase II exit from the pausing period at the TAR element.

3. Tat-dependent antitermination of the transcription at the TAR element is described using the following two reactions:

$$\begin{array}{c} TAR\text{\_}RNApol-II\text{\_}elong+protTat\text{\_}nuc\Leftrightarrow Tat\text{\_}TAR:k_{ass,Tat\text{\_}TAR},k_{dis,Tat\text{\_}TAR}\\ Tat\text{\_}TAR\to DNAunit\text{\_}RNApol-II\text{\_}elong\text{\_}\left(1\right)+protTat\text{\_}nuc:k_{antiterm}. \end{array}$$

The first reaction describes the interaction of the complex <*TAR_RNApol-II_elong*> prone to termination at the TAR element with the nuclear fraction of the Tat protein <*protTat_nuc*> resulting in the formation of the <*Tat_TAR*> complex. The second reaction takes into account the Tat-dependent antitermination leading to the creation of the elongation complex and the release of Tat protein. The parameters *k_ass,Tat_TAR _*и *k_dis,Tat_TAR _*represent the constant rates for association and dissociation of the Tat protein with the TAR element, respectively; *k_antiterm _*is the rate constant of the transcriptional antitermination by Tat protein at the TAR-element.

4. To simulate the delay in the synthesis of 9 kb RNAs, we used the chain of *n_DNAunit _*reactions to describe the transcription elongation from the TAR element to the transcription terminator:

$$\begin{array}{c} DNAunit\text{\_}RNApol-II\text{\_}elong\text{\_}\left(i\right)\to DNAunit\text{\_}RNApol-II\text{\_}elong\text{\_}\left(i+1\right):k_{transcr,elong,i},\\ i=1,...,n_{DNAunit}, \end{array}$$

where *DNAunit_RNApol-II_elong_*(*i*) denotes the number of the elongating complexes at the *i*-th segment of the proviral DNA; *k_transcr,elong,i _*is the transcription elongation rate constant at the *i*-th segment. It is assumed that the lengths of the segments and therefore, the transcription rates are the same.

5. The transcription termination finalizing the elongation of the last (*n_DNAunit_*+1)-th segment of the proviral DNA and the release of the precursor molecule of the nuclear 9 kb RNA <*pre-9 kb-RNA_nuc*> is represented as follows:

$$DNAunit\text{\_}RNApol-II\text{\_}elong\text{\_}\left(n_{DNAunit}+1\right)\to pre-9kb-RNA\text{\_}nuc:k_{transcr,term}$$

with *k_transcr,term _*denoting the transcription termination rate constant of the 9 kb RNA.

6. The maturation of the 9 kb mRNA primary transcript into the mature 9 kb mRNA form <*9 kb-RNA_nuc*> is modelled by:

$pre-9kb-RNA\text{\_}nuc\to 9kb-RNA\text{\_}nuc:k_{modif}$,

where *k_modif _*is the rate constant of the primary 9 kb RNA maturation.

7. Splicing of the 9 kb RNA leading to the formation of 4 kb RNA <4 kb - RNA_nuc> in the nucleus is represented by:

$\begin{array}{c} 9kb-RNA\text{\_}nuc\to 4kb-mRNA\text{\_}nuc:k_{splicing,94}\\ Rev\text{\_}\left(i\right)\text{\_}9kb-mRNA\text{\_}nuc\to Rev\text{\_}\left(i\right)\text{\_}4kb-mRNA\text{\_}nuc:k_{splicing,94},i=1,...,n_{Rev} \end{array}$.

Here *k_splicing,94 _*is the splicing rate constant from 9 kb to 4 kb mRNAs.

8. Alternative splicing of the 4 kb RNA to 2 kb mRNAs which encode the Tat <*2 kb-RNA_Tat_nuc*> and Rev <*2 kb-RNA_Rev_nuc*> proteins is described by

$$4kb-RNA\text{\_}nuc\to \left[\delta_{4,Tat}\right]\cdot 2kb-RNA\text{\_}Tat\text{\_}nuc+\left[\delta_{4,Rev}\right]\cdot 2kb-RNA\text{\_}Rev\text{\_}nuc:\;\;k_{splicing,42}.$$

We denote by *k_splicing,42 _*the rate constant for splicing of 4 kb mRNA to 2 kb; and the parameters *δ_4,Tat_*, *δ_4,Rev _*stand for the fractions of 2 kb RNAs *2 kb-RNA_Tat_nuc *and *2 kb-RNA_Rev_nuc *produced by an alternative splicing of 4 kb mRNA.

9. Transport of 2 kb mRNAs into the cytoplasm is modelled by the equation: $2kb-RNA\text{\_}Xxx\text{\_}nuc\to 2kb-RNA\text{\_}Xxx\text{\_}cyt:k_{transp,2kb-mRNA\text{\_}Xxx},Xxx\in \left\{Tat,Rev\right\}$.

The concentration of 2 kb mRNAs encoding Tat and Rev in cytoplasm is denoted by <2 *kb-RNA_Xxx_cyt*> and *k_transp, 2 kb-RNA_Xxx _*represents the nuclear export rate constant for 2 kb RNA for the Tat and Rev proteins.

10. The synthesis of the Tat and Rev proteins in the cytoplasm is described as a chain of reactions, considering the initiation, elongation and termination of translation: $\begin{array}{c} {}[0]2kb-RNA\text{\_}Xxx\text{\_}cyt\to 2kb-RNA\text{\_}Xxx\text{\_}transl-elong\text{\_}\left(1\right):k_{transl,ini,Xxx}\\ 2kb-RNA\text{\_}Xxx\text{\_}transl-elong\text{\_}\left(i\right)\to 2kb-RNA\text{\_}Xxx\text{\_}transl-elong\text{\_}\left(i+1\right):k_{transl,elong,i},\\ \;\;\;\;\;\;\;\;\;\;\;\;\;\;\;\;\;\;\;\;\;i=1,...,n_{Xxx},\\ 2kb-RNA\text{\_}Xxx\text{\_}transl-elong\text{\_}\left(n_{Xxx}+1\right)\to prot\text{\_}Xxx\text{\_}cyt:k_{transl,term,n_{Xxx}+1},Xxx\in \left\{Tat,Rev\right\}. \end{array}$ Here *k_transl,ini,Xxx _*is the initiation of translation rate constant, *k_transl,elong,i _*is the translation elongation rate constant for *i*-th segment and *k_transl,term,nXxx+1 _*stands for the translation termination rate constant for (*n_Xxx_*+1)-th segment. The first reaction describes the initiation of the Tat and Rev proteins translation in the cytoplasm resulting in the formation of the elongation complex <2 *kb - RNA_Xxx_transl-elong_*(1)>. The next *n_Xxx _*reactions consider the translation elongation for the Tat and Rev proteins. The use of the reactions chain serves to reproduce the delay in the synthesis of Tat and Rev. The set <2 *kb - RNA_Xxx_transl-elong_*(*i*)> characterizes the number of elongation complexes on *i*-th segment of the viral mRNAs. The last reaction describes the translation termination associated with the formation of Tat and Rev. <prot_Xxx_cyt> denotes the amount of the Tat and Rev proteins in the cytoplasm.

11. The transport of the Tat and Rev proteins from the cytoplasm to the nucleus is modelled by the equation:

$$prot\text{\_}Xxx\text{\_}cyt\to prot\text{\_}Xxx\text{\_}nuc:k_{tranp,prot\text{\_}Xxx},Xxx\in \{Tat,Rev\},$$

where <prot_Xxx_nuc> is the abundance of Tat and Rev in the nucleus, and *k_transp,Xxx _*is the protein-specific rate constant for transport from the cytoplasm to the nucleus for Tat and Rev.

12. The formation of the complexes of the Rev proteins with 9 kb RNAs and 4 kb RNAs is described as a sequence of *n_Rev _*reactions resulting in the production of *n_Rev_*-th complex. It is assumed that the complexes of Rev with 9 kb- and 4 kb RNAs can take place both in the nucleus and the cytoplasm. The corresponding set of equations reads:

$$\begin{array}{c} Xkb-RNA\text{\_}yyy+prot\text{\_}Rev\text{\_}yyy\Leftrightarrow Rev\text{\_}\left(1\right)\text{\_}Xkb-RNA\text{\_}yyy:k_{ass,Rev\text{\_}\left(1\right)\text{\_}X\text{\_}yyy},k_{dis,Rev\text{\_}\left(1\right)\text{\_}X\text{\_}yyy}\\ Rev\text{\_}\left(i\right)\text{\_}Xkb-RNA\text{\_}yyy+prot\text{\_}Rev\text{\_}yyy\Leftrightarrow Rev\text{\_}\left(i+1\right)\text{\_}Xkb-RNA\text{\_}yyy:k_{ass,Rev\text{\_}\left(i+1\right)\text{\_}X\text{\_}yyy},k_{dis,Rev\text{\_}\left(i+1\right)\text{\_}X\text{\_}yyy},\\ \;\;\;\;\;\;\;\;\;\;\;\;\;\;\;\;\;\;\;\;\;\;\;\;\;\;\;\;\;\;\;\;\;\;\;\;\;\;\;\;\;\;\;i=1,...,n_{Rev}-1,X\in \left\{9,4\right\},yyy\in \{nuc,cyt\}. \end{array}$$

The variables and the parameters have the following meaning: <*Rev*_(*i*)_*X kb*-*RNA_yyy*> is the number of *i*-molecules containing complexes of the Rev proteins with either 4 kb- or 9 kb RNAs in the nucleus and in the cytoplasm, respectively; *k_ass,Rev__*_(*i+*1)_, *k_dis,Rev__*_(*i+*1) _- are the association- and dissociation rate constants for Rev binding with 4 kb RNA and 9 kb RNA at the stage of the (*i*+1)- meric complex formation in the nucleus or the cytoplasm.

13. Rev-dependent transport of 9 kb RNA and 4 kb RNA from the nucleus <*Rev*_(*i*)_*X kb*-*RNA_nuc*> to the cytoplasm <*X kb*-*RNA_cyt*> followed by the release of the Rev protein in the nucleus (nuclear pore complex) and in the cytoplasm is described by the equations

$$\begin{array}{c} Rev\text{\_}\left(i\right)\text{\_}Xkb-RNA\text{\_}nuc\to Xkb-RNA\text{\_}cyt+\left[\gamma_{i}\right]\cdot prot\text{\_}Rev\text{\_}nuc+\left[i-\gamma_{i}\right]\cdot prot\text{\_}Rev\text{\_}cyt:k_{transp,X,i},\\ \;\;\;\;\;\;\;\;\;\;\;\;\;\;\;\;\;\;\;\;\;\;\;\;\;i=1,...,n_{Rev,X}-1,X\in \{9,4\}. \end{array}$$

Here, *k_iransp,X,i _*stands for the rate constant of the nuclear export of the *i*-meric complex of Rev with 9 kb- or 4 kb RNA; *γ_i _*is the fraction of the Rev protein, released from the *i*-meric complex in the nuclear pore complex compartment; (*i*-*γ_i_*) is the fraction of Rev, released from the *i*-meric complex in the cytoplasm.

14. The degradation of the 9 kb-, 4 kb-, 2 kb RNAs in the nucleus and cytoplasm is described by the following first order reactions:

$$\begin{array}{c} Xkb-RNA\text{\_}yyy\to \emptyset :k_{degr,Xkb-RNA\text{\_}yyy},\\ 2kb-RNA\text{\_}yyy\to \emptyset :k_{degr,2kb-RNA\text{\_}yyy},\\ Rev\text{\_}\left(i\right)\text{\_}Xkb-RNA\text{\_}yyy\to \left[\gamma_{i}\right]\cdot prot\text{\_}Rev\text{\_}nuc:k_{degr,Rev\text{\_}\left(i\right)\text{\_}Xkb-RNA\text{\_}yyy},\\ prot-Xxx\text{\_}yyy\to \emptyset :k_{degr,prot-Xxx\text{\_}yyy},\\ \;\;\;\;\;\;\;\;\;\;\;\;\;\;\;\;\;\;i=1,...,n_{Rev,X}-1,X\in \left\{9,4\right\},Xxx\in \left\{Tat,Rev\right\},yyy\in \{nuc,cyt\}. \end{array}$$

Here *k_degr,X kb-RNA_yyy _*are the 9 kb- and 4 kb RNAs degradation rate constants in the nucleus and cytoplasm; *k_degr,prot_yyy _*are the protein specific degradation rate constants for the Tat and Rev proteins; and *k_degr,Rev_(i)-RNA_yyy _*stand for the degradation rate constants for 9 kb and 4 kb RNAs in the nucleus and cytoplasm in the *i*-meric complex with the Rev protein.

### Assembly of the model from basic subsystems and the computational method

The overall change rates for all the species concentrations considered in the model are calculated by summing up the local rates over all elementary subsystems which consider the respective reactants. To solve numerically the initial value problem for the model equations we used the Gear's method based on backward differentiation formulae [37] implemented in Fortran. The source code is available upon request to the first author.

### Estimation of the model parameters

The estimation of the model parameters was carried out by using published experimental data or some fundamental biological considerations when the specific data were not available.

***Transcription: ***The transcription elongation rate in eukaryotic cells ranges from 25 to 60 nucleotides/sec [38-40]. For HIV-1 a similar estimate of about 33 nucleotides/sec was obtained using the reporter construct with 70 copies of it integrated at specific transcription site of the viral genome [41,42]. We assumed that the basal transcription rate starting from the HIV-1 promoter in non-activated cells is about 40 nucleotides/sec, whereas the transcription initiation is about 0.25 events per minute.

The exit rate of RNA-Pol-II from transcription elongation pausing is described by parameter *k_delay_*. We estimated the parameter value to be *k_delay _*= 1 min^-1^, assuming that the duration of the pausing is about 1 min. The underlying arguments are based on the view that the pausing time should be longer then the time of the transcription through the TAR element by RNA-polymerase II (RNApol-II) without the pausing, which ranges from 1 to 2 seconds. The later estimate results from the base length of the TAR element being 59 nucleotides and the elongation rate of about 25-60 bases/sec [38,40]. In the absence of the Tat protein, RNApol-II located at the TAR element can spontaneously leave the pausing and either continue the transcription, or terminate it. We assumed the ratio of 1:100, i.e. in the absence of Tat the pausing leads to the transcription termination for about 99 RNApol-II molecules out of one hundred. The RNA polymerase II can also exit the pausing and continue the transcription in the presence of the Tat protein due to its interaction with the secondary structure of the TAR element on the synthesized RNA. The availability of Tat in the nucleus activates the synthesis of 9 kb RNA by up to 25- to 100-fold [23,43,44].

The antitermination efficacy in the model is described by the parameter *k_Tat-antiterm,TAR_*. We used the estimate *k_Tat-antiterm,TAR _*= 60 min^-1^. The parameters *k_ass,Tat-TAR _*and *k_dis,Tat-TAR_*, represent the binding and dissociation rate constants for the Tat protein with the secondary structure of the partially synthesized RNA at the TAR-element region, respectively (see Table 1). The published data provide the estimate for the ratio *K_dis,Tat_*=*k_dis,Tat-TAR_*/*k_ass,Tat-TAR _*with the range 100-400/μM [45,46]. In the computations we assumed the dissociation rate constant to be *k_dis,Tat-TAR_*= 1 min^-1^, which was derived using the values *K_dis,Tat _*= 100/μM and the volume of the nucleus of 100 μm^3 ^(calculated from [47]).

***Splicing: ***According to the experimental data [38,48,49], the splicing of the pre-mRNAs takes place during the transcription with the characteristic time of about 5 to 10 min and is not dependent on the introns size.

***Transport: ***The transport of 2 kb mRNA from the nucleus to the cytoplasm is carried out by endogenous cellular mechanisms. According to the available data [50,51], the active transport through the nuclear pore is a relatively fast process (10 to 100 molecules per the pore per second) and therefore, can be described as a single event via the monomolecular reaction equation.

The transport of 9 kb- and 4 kb RNAs out of the nucleus is mediated by Rev-dependent mechanism. According to the available data [28,52,53] the binding of the Rev protein to the RRE site of the intron-containing HIV-1 mRNA takes place sequentially and the formed oligomeric complex Rev/RRE can contain up to 12 molecules of the Rev protein.

The transport of Tat and Rev back to the nucleus is mediated by the endogenous cellular mechanisms through the nuclear localization sequence (NLS) signal. The transport kinetics of Tat was characterized by the kinetics of heterologous protein bound to the Tat NLS under in vivo conditions [54]. The experimental data suggest that the protein specific rate constant of the nuclear import is 0.303 ± 0.099 min^-1^. We assumed in the model that the Tat and Rev nuclear import rate constants are similar and of 0.347 min^-1^.

***Translation: ***The published data [55,56] suggest that the average ribosome density per codon in eukaryotic cells is 0.017. Taking into account the length of Tat/Rev mRNA of about 2000 bases, the number of available ribosomes at this segment can be estimated to be about 11. The translation elongation rate of mRNA depends on the initiation rate. In the model we assumed the initiation rate to be about 10 per min, which suggests that the translation elongation rate constant is about 30 nts/sec.

***mRNA stability: ***The data by [57] suggest that the stability of the HIV-1 mRNA in both compratments (the nucleus and the cytoplasm) is about the same with the half-life for the Tat mRNA being 4 to 5 hours and that of Rev ranging from 4 to 13 hours [58,59].

***Stability of proteins: ***The half-life of Rev is assumed in the model to be about 4 hours and 16 hours in the cytoplasm and the nucleus, respectively [60].

The parameters of the mathematical model used in computations are summarized in Table 1.

### Basic model of the Tat-Rev regulatory circuit

The basic model of the Tat-Rev regulatory circuit is composed of subsystems referred to in Table 1 as 1-11, 12 (for the nucleus) and 13. It is assumed that Rev protein binding to the RRE element on 9 kb and 4 kb RNAs terminates their splicing to the 2 kb mRNA (Figure 1), in agreement with the available data [61-63].

As the basic model neglects the formation of the complexes of the *de novo *synthesized Rev proteins with the 9 kb- and 4 kb RNA molecules in the cytoplasm, subsystem 12 is applied to the nuclear mRNAs only.

**Figure 2:**
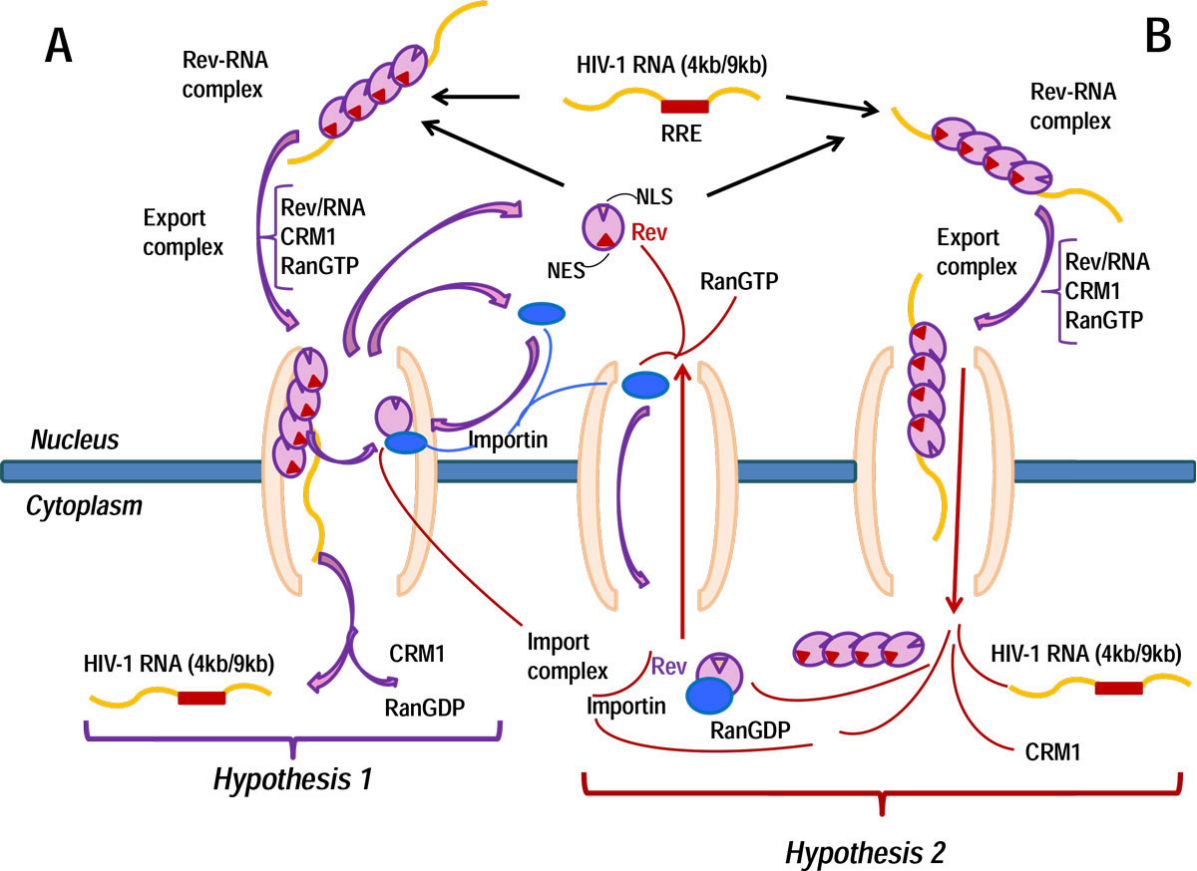
**Schematic representation of Rev-mediated transport of intron-containing 4 kb and 9 kb RNAs and of the Rev protein recycling**. (A) - hypothesis 1 for the Rev recycling at the nuclear pore complex; (B) - hypothesis 2 for the Rev recycling in the cytoplasm. RRE - Rev binding site on 4 kb and 9 kb RNAs; Crm1, RanGTP, RanGDP, Importin - are the major cellular factors mediating the RNA export and import of RNA.

There is no clear view on the re-cycling (import/export cycle) mechanisms for the Rev protein. It is considered that the release of Rev from the nuclear export complex can take place in the cytoplasm (hypothesis 1) [19] as well as in the nucleus (hypothesis 2) [35,36]. The scheme of the Rev recycling for the two hypotheses is shown in Figure 2. We analyze the implications of hypotheses 1 and 2. The basic model implements the first hypothesis, i.e. the Rev protein is considered to be released from the nuclear export complex in the nuclear pore compartment to return to the nucleus. Therefore, in subsystem 13 zero values of the following parameters are used γ*_i _*= 0, *i *= 1,...,12. The analysis of the second hypothesis would require non-zero values to be assigned to γ*_i_*.

## Results

### Synthesis of viral components in non-activated and activated cells

Consider a non-activated cell with one proviral HIV-1 genome. The basal level of the transcription initiation at the viral promoter site is about 0.25 per min (see Section on Parameter estimation). The model computations predict that for the parameter values listed in Table 1 a steady state pattern is established.

**Figure 3:**
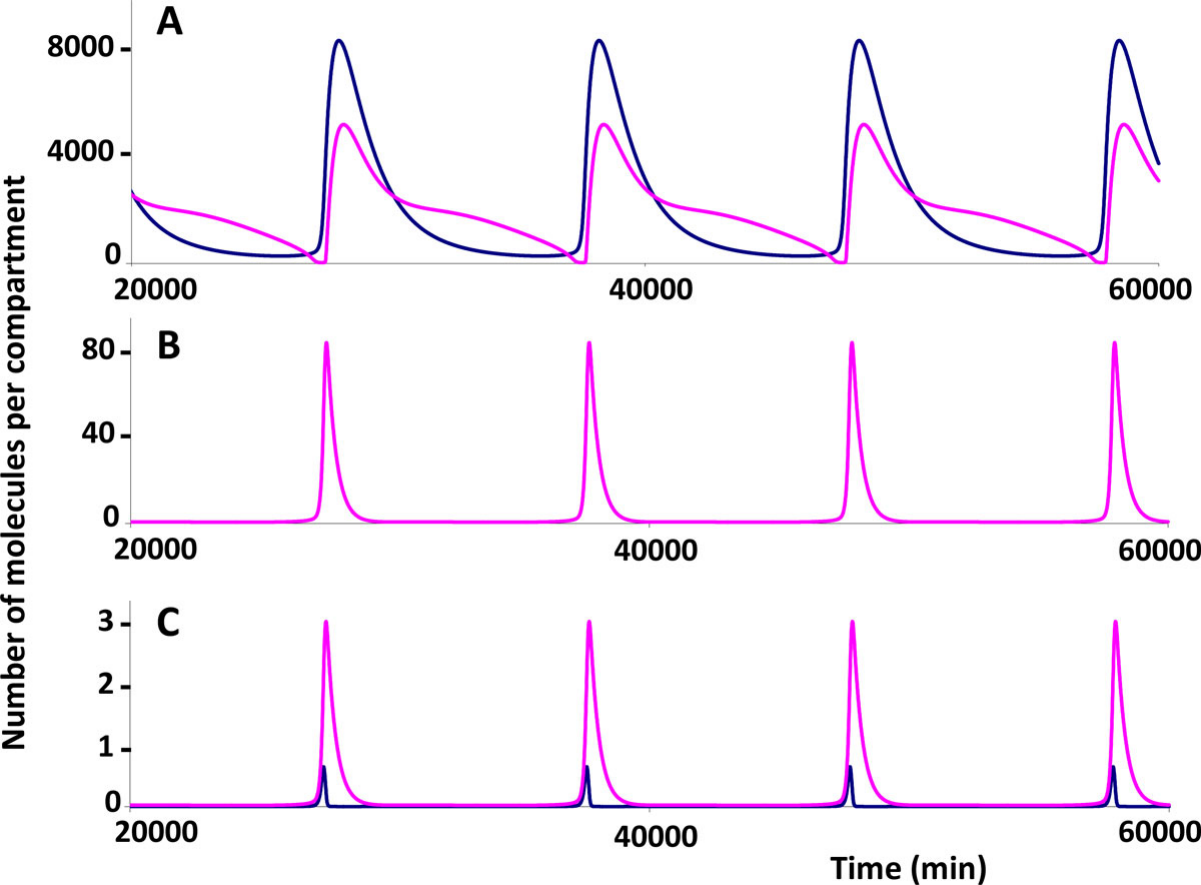
**Kinetics of the viral RNA and proteins synthesized in an activated cell with one provirus copy**. *А *- the abundance of free Tat molecules (not bind to RNA at the TAR element) (blue) and Rev molecules (not bind to 9 kb RNA and 4 kb RNA) (pink) in the nucleus; *B *- the abundance of free Tat and Rev proteins in the cytoplasm (their kinetics is identical as the corresponding model parameters for these two proteins are identical and the nuclear export of Rev to the cytoplasm is not considered); *C *- the abundance of 2 kb RNA molecules in the nucleus (blue) and cytoplasm (pink) encoding the Tat and Rev proteins.

According to [64], in an activated cell the concentration of active NF-κB molecules sets up to the level that is enough to increase the initiation of transcription rate at the viral promoter site to about 25 initiations per min as estimated in [65]. The model computations predict an oscillatory functioning mode for the corresponding parameter values (Table 1). The dynamic details of the oscillatory pattern are shown in Figure 3.

It should be noted that the oscillatory pattern in Figure 3 appears as a limit cycle. The period of the cycle is rather long being of more than 150 hours. The amplitude of the oscillations in the concentrations of the Tat and Rev proteins and the 2 kb RNAs encoding Tat and Rev over one period is quite significant. The simulations further predict that the 2 kb RNA molecules are present in trace amounts and are amenable to detection during a rather short time window of about 10 hours as compared to the cycle period.

The above solutions were computed under the assumption that the Rev-regulated export of 9 kb RNA and 4 kb RNA from the nucleus to the cytoplasm does not lead to the exit of the Rev protein from the nucleus to the cytoplasm, which is in agreement with the hypothesis of the Rev recycling in the nuclear pore complex [36]. In addition, we did not consider the formation of the complexes of the *de novo *synthesized Rev proteins with the 9 kb RNA and 4 kb RNA molecules in the cytoplasm compartment. As the corresponding processes are poorly understood for real systems, we examine their impact on the kinetics of the viral molecules synthesis in the next section.

### Impact of the nuclear export of the HIV-1 Rev protein to the cytoplasm on the kinetics of viral components synthesis in an activated cell

There are some data suggesting that the Rev protein bearing the nuclear localization sequence (NLS) and the nuclear export sequence (NES) can shuttle between the nucleus and the cytoplasm [26,27]. The available data on the mechanisms of the Rev-mediated transport of the intron-containing viral RNA [19,36] are not definitive enough to select one out of the two hypotheses on the Rev recycling mechanism.

As indicated in the previous section, in the basic model we consider the hypothesis on the Rev recycling in the nuclear pore complex as the preferred one for which most of the analysis is made [36]. However, the possibility of Rev leaving the nucleus for the cytoplasm of the cell can not be completely excluded. In addition, there are no reasons to completely rule out the interaction of the Rev protein with 9 kb RNA and 4 kb RNA in the cytoplasm.

To examine the impact of the above processes on the dynamics of viral components in the infected cell, we started with the analysis of the model in which either complete or partial export of the Rev protein in the complex with the viral 9 kb and 4 kb RNAs from the nucleus to the cytoplasm is considered. The maximum number of the Rev protein molecules that can bind to one viral 9 kb- or 4 kb RNA and be further exported to the cytoplasm from the nucleus is estimated of about 12 [51,52]. The interaction of the Rev protein with 9 kb- and 4 kb RNAs in the cytoplasm was not considered in the model.

**Figure 4:**
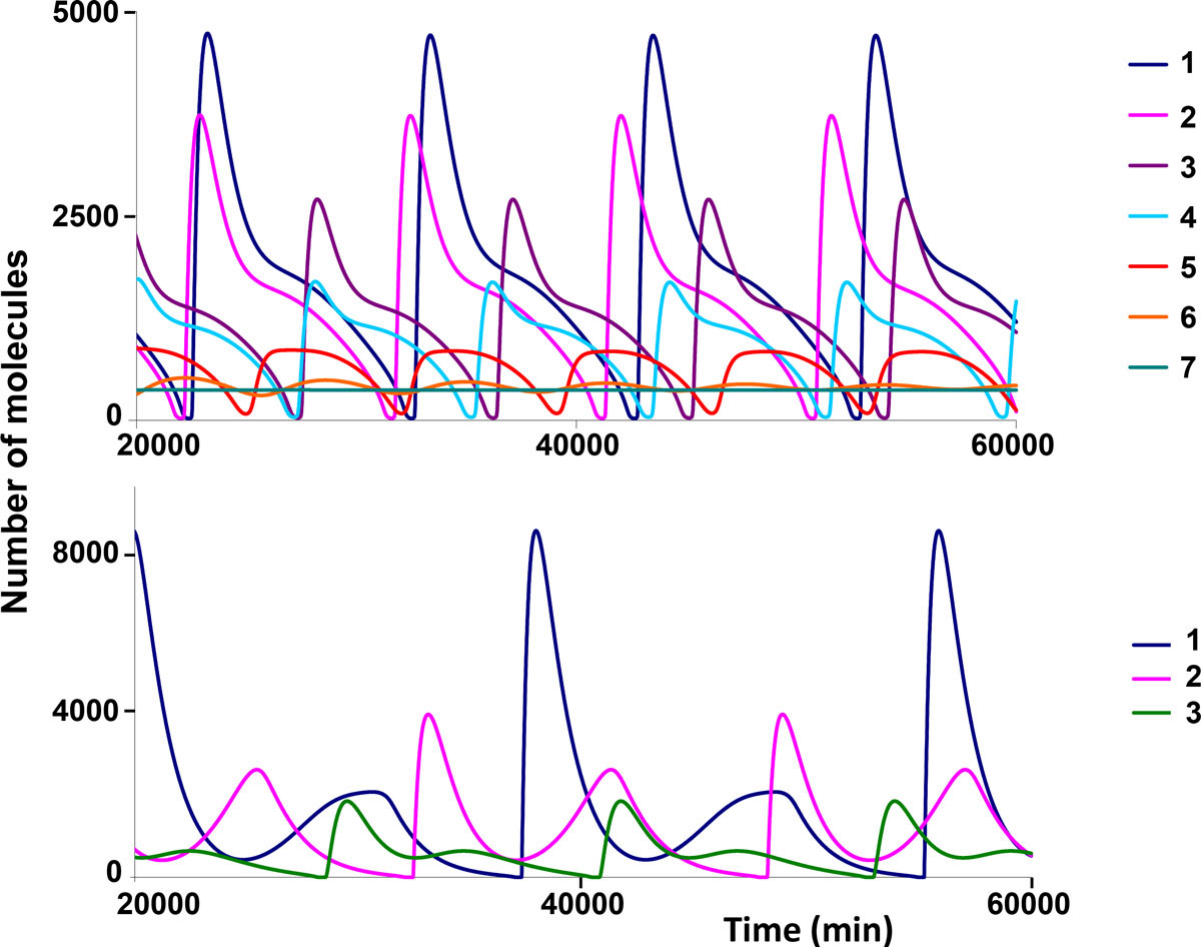
**Dependence of the intracellular Rev protein kinetics on the re-export scale from the nucleus to the cytoplasm**. The upper panel shows the model simulations in which the interaction of the Rev protein with the 9 kb and 4 kb RNAs in cytoplasm is excluded. The presented curves were computed for various numbers of the Rev protein molecules exported from the nucleus to the cytoplasm due to the transport of the multimeric complexes <Rev_(i)_9 kb-RNA_nuc> and the <Rev_(i)_4 kb-RNA_nuc> from the nucleus to the cytoplasm, i = 1,...,6. Curve 1 corresponds to the scenario when all the Rev protein molecules in the *i*-meric oligomer complex are released in the nuclear pore complex so that none of the Rev protein molecules enters the cytoplasm; curves 2,3,4,5,6, and 7 represent the Rev protein kinetics characterized by the export of 1,2,3,4,5 and 6 molecules of the Rev protein to the cytoplasm with the *i*-meric complex, respectively. The rest of the molecules of the above complexes as well as of the complexes with a lower number of the Rev proteins are released in the nuclear pore complex to remain in the nucleus. The lower panel shows the model simulations corresponding to the scenario in which a complete nuclear export of the Rev molecules to the cytoplasm and the interaction of Rev with the 9 kb and 4 kb RNAs in the cytoplasm take place. The effect of *k_dis _*is shown with curve 1 corresponding to *k_dis _*= 8.4 min^-1^, curve 2 to *k_dis _*= 20 min^-1 ^and curve 3 to *k_dis _*= 50 min^-1^. The vertical axis specifies the number of unbound Rev protein molecules in the nucleus.

The model computations predict that an increase in the fraction of the Rev molecules exported from the nucleus to the cytoplasm as a part of the oligomer complex with the intron-containing HIV-1 RNA leads to the transition from the periodic solution to a steady state (Figure 4). The oscillatory dynamics persists as long as the number of the Rev molecules exported to the cytoplasm is less than 5 (out of 12), whereas larger numbers result in a steady-state behavior. It should be noted that an increase in the fraction of exported RNA molecules leads to a decrease of the amplitude and the period of the oscillations (Figure 4, upper panel).

However, the negative effect of the nuclear re-export of the Rev protein on the oscillatory replication dynamics can be removed by considering the interaction of the Rev protein with 9 kb RNA and 4 kb RNA in the cytoplasm, similar to the processes taking place in the nucleus. The corresponding simulation results for the complete re-export case (i.e., all the Rev molecules in the complex with 9 kb RNA and 4 kb RNA are transported to the cytoplasm from the nucleus) are shown in Figure 4 (lower panel). One can see that taking account of the complex formation of Rev with 9 kb RNA and 4 kb RNA in the cytoplasm restores the oscillatory mode. The restoration of the cycling behavior takes place at a lower stability of the complex in the cytoplasm as compared to the nucleus.

It should be noticed that taking the account of (1) the re-export process of the Rev protein and (2) the complex formation of the Rev molecules with 9 kb RNAs and 4 kb RNAs in the cytoplasm leads to a marked increase of the period of the oscillatory patterns in the virus replication. For example, in simulation shown in Figure 3 with no consideration given to the re-export of the Rev protein and the complex formation between the Rev molecules and the 9 kb RNAs and 4 kb RNAs in the cytoplasm, the period of the oscillations is about 160 to 170 hours. However, in simulations displayed in Figure 4, the cycle duration ranges from 200 to 400 hours.

### Impact of provirus copy number on the viral components synthesis kinetics in an activated cell

In the previous sections we studied the model predictions for the case of one provirus copy (model parameter *proV *= 1) in the infected cell. In this case, in the absence of the Rev protein re-export from the nucleus and the complex formation of the Rev protein with the 9 kb RNAs and 4 kb RNAs in the cytoplasm, the steady state pattern of the virus components replications changes to an oscillatory dynamics if the activity of the transcription initiation exceeds the threshold of 5 events per min. Notice that the above value is still below the maximum transcription initiation rate which is observed in activated cells as the data suggests [66]. However, it cannot be excluded that the transcription initiation activity in some cells remains below the threshold. The model predicts that in these cells with one provirus copy a steady state mode of the viral components synthesis is established. It is known that the infected cell can harbor more than one provirus [67]. As the overall efficiency of the transcription initiation increases with an increase of the proviral copy number, in the cell which harbor more than one provirus the total transcription initiation rate can become larger than the above threshold thus leading to the oscillatory replication dynamics. Given that the total intensity of the transcription initiation according to the model is the product of $k_{transcr,ini}\times proV$, then for the transcription initiation parameter value *k*_ini _= 1 events/min the periodic regime takes place when the number of proviral genomes per cell was six or more, for the transcription initiation parameter *k*_ini _= 2 events/min - three or more proviral genomes (to ensure $k_{transcr,ini}\times proV$≥6), etc.

### Impact of the abundance of the Rev protein in the complexes with 9 kb RNA and 4 kb RNA on the viral components synthesis kinetics in an activated cell

**Figure 5:**
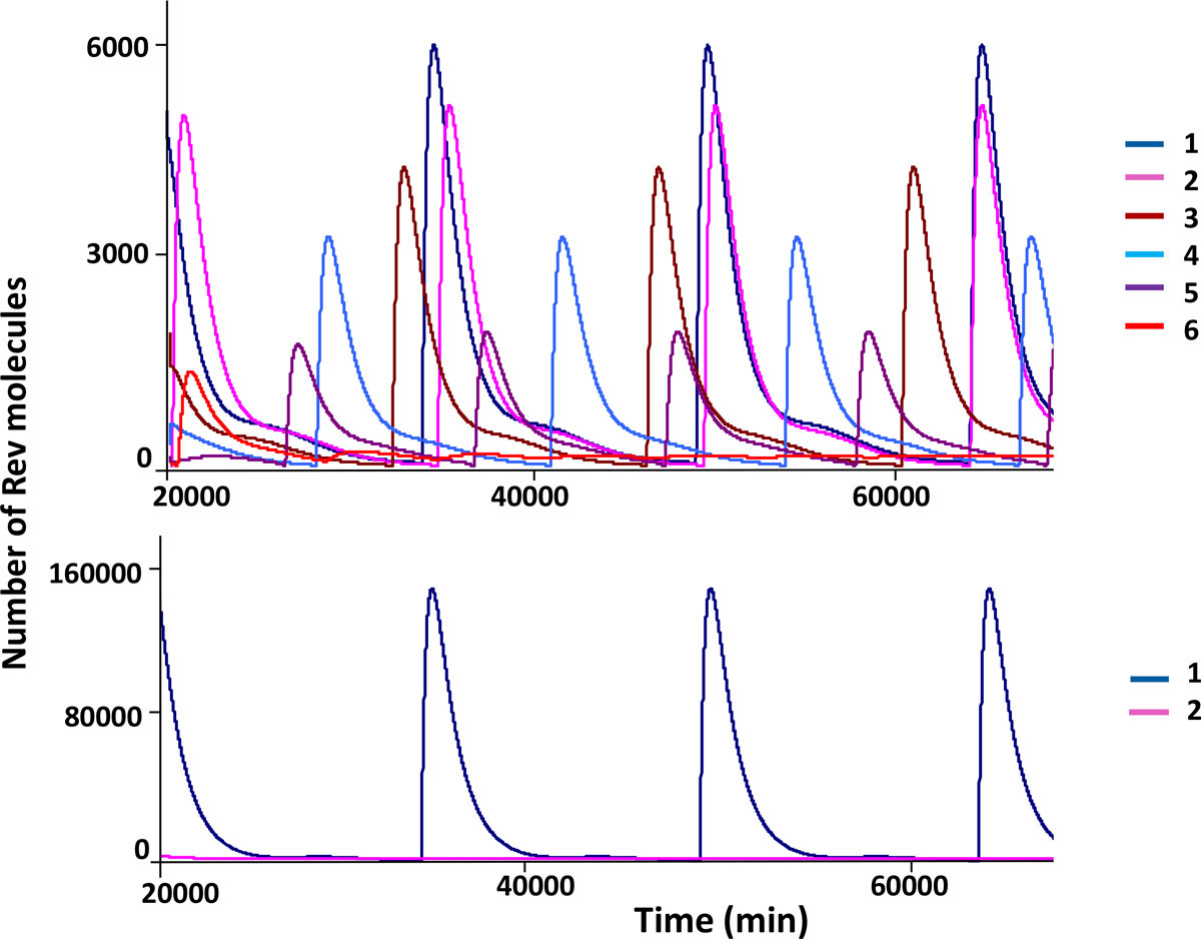
**Kinetics of the Rev protein in the cell in relation to the oligomerization level in the complex with intron-containing viral mRNA and the provirus copy number in the genome**. The model simulations in the absence of the Rev protein re-export from the nucleus and the complex formation of the Rev protein with the 9 kb RNAs and 4 kb RNAs in the cytoplasm. Upper panel corresponds to one proviral copy. Curve 1 corresponds to *n_Rev _*= 12, curve 2 is for *n_Rev _*= 10, curve 3 is for *n_Rev _*= 8, curve 4 is for *n_Rev _*= 6, curve 5 is for *n_Rev _*= 4, curve 6 is for *n_Rev _*= 3. Lower panel corresponds to 10 proviral copies. Curve 1 is for *n_Rev _*= 3 and curve 2 is for *n_Rev _*= 2. The vertical axis specifies the abundance of the free Rev molecules in the nucleus.

It has been established that the Rev protein can bind to the HIV-1 9 kb RNAs and 4 kb RNAs on the RRE site [68]. The number of the proteins molecules that bind to one RNA molecule can be up to n*_Rev _*= 12 [52,53]. This value was used in the simulations presented above. The model predictions for different values of the parameter *n_Rev _*= 12, 10, 8, 6, 4, 3 are shown in Figure 5 (upper panel). One can clearly see that the oscillatory dynamics of the viral components synthesis is sensitive to the value of the above parameter and for n*_Rev _*< 4 the systems shifts to a steady state mode. However, if for n*_Rev _*= 3 the proviral copy number parameter is increased up to 10, the oscillatory behavior is restored (Figure 5, upper panel, curve 1). For n*_Rev _*= 2 the viral components production kinetics returns to a steady state mode (Figure 5, lower panel, curve 2). The oscillatory dynamics can be regained by an increase of the number of proviruses to extremely high values which are beyond the physiological limits. Indeed the model displays a steady state solution for the *proV *= 100 copy/cell and only for the copy number close to *proV *= 150 copy/cell a stable periodic solution is predicted. For the lowest value *n_Rev _*= 1 the oscillatory solutions were not revealed.

Overall, the oscillatory dynamics of the viral components synthesis in an activated cell is robustly reproduced for the following range of the relevant parameter: 4≤ *n_Rev_*≤12. For *n_Rev _*= 3 the dynamics depends on the number of proviruses: for *proV *= 1 a steady state behavior takes place whereas for *proV *= 10 an oscillatory regime is observed. For *n_Rev _*= 2 the qualitative behavior is similar to the case *n_Rev _*= 3, however, for the emergence of the periodic regime much higher number of the proviral copies is required going beyond the physiological values. No oscillatory dynamics was observed for *n_Rev _*= 1.

### Impact of the parameters of the transcription antitermination at the TAR-element on the kinetics of the viral components synthesis in an activated cell

In the proposed model the antitermination of transcription is described by subsystem 3 (see the «Elementary subsystems of the model» section). The process is characterized by four parameters: the rate constant for the exit of RNA polymerase II from the pausing at TAR-element, *k_delay_*; the rate constant of the antitermination efficacy, *k_Tat-antiterm,TAR_*; the forward and reverse rate constants of the Tat protein binding to the secondary structure at the TAR-element, *k_ass,Tat-TAR _и k_dis,Tat-TAR_*, (Table 1). The published data suggest that an estimated ratio *K_dis,Tat _*= *k_dis,Tat-TAR_*/*k_ass,Tat-TAR _*should be around 10 nM [45]. Therefore, the antitermination system is described in the model by three independent parameters out of four. The results presented in the above section were obtained for the specific values of these parameters given in Table 1. To assess the impact of the parameters characterizing the transcription antitermination at the TAR-element on the model dynamics, a set of simulations was carried out with the parameter values ranging as given below:

$k_{delay}=0.25-10\;{\text{min}}^{-1},k_{Tat-antiterm,TAR}/k_{delay}=\;1-60,k_{dis,Tat-TAR}=0.1-32\;{\text{min}}^{-1}.$

The obtained results suggest that the oscillatory mode is realized in a rather broad range of changes in these parameters. At the same time it can be noted that there is exists a definite relationship between the values of variable parameters. In particularly when the values of rate constant for the exit of RNA polymerase II from the pausing at TAR-element, (*k_delay_*) decrease the oscillatory regime is realized at the range of higher the values of constant rates for dissociation of the complex Tat protein with TAR-element (*k_dis,Tat-TAR_*).

### Impact of the parameters of oligomerization of the Rev protein on the RRE site of the intron-containing RNA and parameters of the complex transport on the viral components synthesis kinetics

Studies of the interaction of the Rev protein with the RRE site on the 9 kb and 4 kb HIV-1 RNAs indicate that the forward and reverse reaction rate constants of the consecutive binding of four Rev molecules are close but somewhat different [28]. Therefore, we set the rate constants for the first 4 sequential reactions as suggested in [28], and for the rest reactions (from 5 to 12) the parameters were assumed to be equal to that of the 4^th ^reaction.

**Figure 6:**
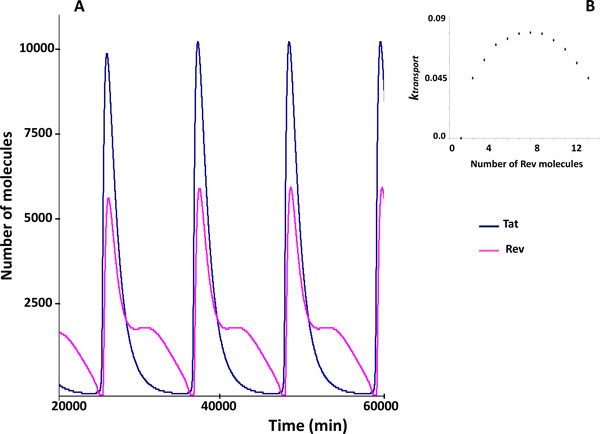
**Dynamics of the accumulation of the Tat and Rev proteins in the nucleus depending on the REV_n_-RNA complex transport parameters** (A). (B) The transport rate constants of the intron-containing HIV-1 RNA depend on the number of the Rev molecules (*n*) in the oligomer complexes <*Rev*_(*n*)_*9 kb*-*RNA_nuc*> and <*Rev*_(*n*)_*4 kb*-*RNA_nuc*>. This dependence was used for the model simulations.

There are data indicating that the functional efficacy of the nuclear export complex of Rev with 9 kb- and 4 kb RNA depends on the degree of oligomerization and the complexes containing 6 to 8 Rev molecules are the optimal ones [52,53,68]. Based on this observation we assumed that the dependence of the transport rate constant of the 9 kb- and 4 kb RNA on the number (*n*) of the Rev molecules in the complex is a convex unimodal function with its maximum value at *n *= 7 (Figure 6b).

The model simulations with the above defined parameters for the oligomerization of the Rev protein on the RRE site of the 9 kb- and 4 kb RNA and the complexes transport rate constant predict an oscillatory kinetics of the Tat and Rev proteins synthesis (Figure 6a).

### Impact of the 2 kb RNA translation initiation efficacy on the kinetics of the viral components synthesis in an activated cell

In previous sections the modelling was based upon the assumption that for the Tat-Rev mediated regulation of HIV-1 replication the translation initiation rate constants for 2 kb RNAs encoding the Tat and Rev proteins are about $k_{transl,ini,Xxx}=10\;\;\text{ini/min},Xxx\in \left\{Tat,Rev\right\}$, respectively. However, the specific values of the parameters for HIV-1 are not known. Therefore, we computed the model solutions for a set of values of the parameter $k_{transl,ini,Xxx}=10\;\;\text{ini/min},Xxx\in \left\{Tat,Rev\right\}$, as follows: 1, 2, 4, 8, 16, 32, 64 ini/min. For the whole range of the translation initiation rate constants the oscillatory dynamics of the viral components synthesis was preserved.

### Impact of the Rev-mRNA stability on the kinetics of the viral components synthesis in an activated cell

**Figure 7:**
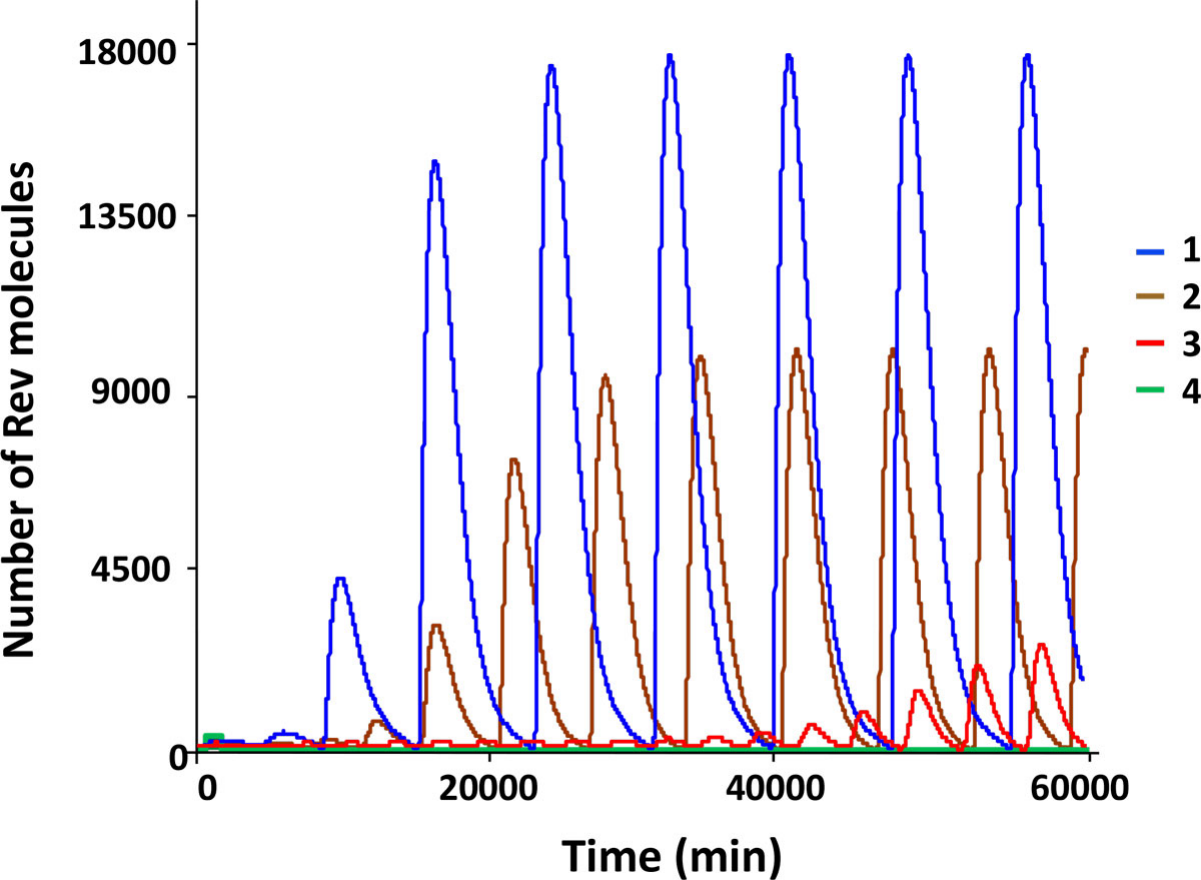
**Impact of the Rev mRNA stability on the kinetics of the viral components synthesis in an activated cell**. The curves are distinguished by the parameters values: curve 1 - *k *= 0.0009 min^-1^, curve 2 - k = 0.0014 min^-1^, curve 3 - k = 0.0015 min^-1^, curve 4 - k = 0.0016 min^-1^, *k *= $k_{degr,2kb\text{\_}RNA\text{\_}Rev\text{\_}yyy}$,yyy∈{nuc,cyt}. Simulations for the version of the model in which the Rev-dependent transport of 9 kb RNA and 4 kb RNA from the nucleus to the cytoplasm leads to the release of the RNA bound Rev proteins into the free cytoplasmic fraction. However, the interaction of Rev with 9 kb- and 4 kb RNAs in the cytoplasm does not take place. The simulations were performed with the model parameter values listed in Table 1.

In the above computational experiments with the mathematical model of the Tat-Rev regulation of HIV-replication, the half-life of mRNA encoding the Rev protein was assumed to be about 4 hours. This estimate was obtained from the work by Felber et al. [58]. However, recent studies suggest that the Rev-mRNA half-life is about 13 hours [59]. We examined the influence of the parameters $k=k_{degr,2kb\text{\_}RNA\text{\_}\text{Re}\;v\text{\_}yyy},yyy\in \left\{nuc,cyt\right\}$ on the dynamics of the viral components synthesis in an activated cell (Figure 7). For simulations we used the version of the model in which the Rev-dependent transport of the 9 kb RNA and 4 kb RNA from the nucleus to the cytoplasm leads to the release of the Rev molecules to the cytoplasm as free molecules (i.e., the re-cyclization of the Rev protein in the cytoplasm takes place). We did not consider the binding of the Rev protein to 9 kb RNA and 4 kb RNA in the cytoplasm. The justification for considering this scenario is related to the observation that the version of the model (with the Rev protein cytoplasmic re-cyclization included) with the parameter values given in Table 1, predicts a steady state dynamics, whereas the model version allowing for the re-cyclization of the Rev protein at the nucleus pore complex shows an oscillatory behavior for the same parameter values.

It turned out that in the range [0.0009, 0.0029], min^-1^, specifying the lower and upper limits for the parameter values estimated from the available data [58,59], the Hopf bifurcation takes place for 0.0015<***k***<0.0016 at which the steady state becomes unstable and the limit cycle emanates (Figure 7). The limit cycle exists for *k *in the range [0.00009, 0.0012] (indicating a hysteresis phenomenon), and for ***k***<0.00009 min^-1 ^the stable steady state is realized.

Therefore, the computational analysis of the model showed that when the re-cyclization of the Rev protein in the cytoplasm takes place and the Rev protein does not interact with the 9 kb- and 4 kb mRNA in the cytoplasm then the oscillatory dynamics is observed for a stable complex Rev-mRNA, i.e., for the values of the respective parameters $k=k_{degr,2kb\text{\_}RNA\text{\_}\text{Re}\;v\text{\_}yyy},yyy\in \left\{nuc,cyt\right\}$in the range [0.00009, 0.0012]. For high degradation rate of the Rev-mRNA complex of about 0.0029 min^−1^, the oscillatory behavior is realized only when the rec-cyclization of the Rev in the nuclear pore complex is considered in the model.

It should be noted, that in the previously published models [30-32,34] only a single value of the parameter *k *was used, i.e. 0.0029 min^-1^, and the Rev re-cyclization mechanism was described in accordance with the cytoplasm based Rev re-cyclization hypothesis. As our model suggest that this should result into a steady state mode, it is not surprising that the oscillatory regime has not been predicted.

In general, the computational results suggest the following:

1) The parameter space region corresponding to the oscillatory dynamics is rather large;

2) The parameters are not independent with respect to their impact on the HIV-1 replication system oscillatory behavior;

3) An increase in the proviral copy number extends the parameter region in which an oscillatory dynamics of the virus proteins synthesis takes place;

4) The true values (i.e., the biologically consistent ones) of the above parameters may well belong to the oscillatory domains of the model parameters space.

## Discussion

In this study we developed the mathematical model of Tat-Rev-dependent regulatory circuit for HIV-1 replication consisting of a positive feedback loop for the viral Tat protein mediated regulation via the antitermination on the TAR element of the proviral DNA (see, the review [19]) and of a negative regulatory feedback loop via the Rev protein mediated repression of the intron-containing viral mRNA splicing [29]. It is known that the presence of both the positive- and negative regulatory feedback loops is a prerequisite for the emergence of complex dynamical behaviors of the system, with an oscillatory dynamics representing one of them [13-17].

Indeed, the analysis of the model revealed its high potential with respect to the generation of oscillatory dynamics that was essentially dependent on the Rev protein re-cyclization mechanism, the stability of its mRNA and the interaction parameters of the Rev protein with the RRE site on the intron-containing RNA. These processes have been identified and described since only few years ago [28,36,59]. Therefore, it is not surprising that in the existing models of the HIV-1 life cycle [30-34] based on more earlier experimental studies, the existence of an oscillatory behavior in the virus replication was not revealed.

Taking into account that the parametric domain corresponding to the oscillatory mode of HIV-1 replication is quite large, we hypothesize that the predicted phenomenon is not a just a modelling artefact but can take place under certain conditions in the infected cell. One of the indirect indications in favor of this hypothesis could be the ability of HIV-1 to establish a long-term persistent production of the infectious particles in humans [69]. Although HIV replication occurs both in CD4 + T-lymphocytes and macrophages, our results on modeling the HIV intracellular ontogeny should be considered in the context of the macrophage infection. Indeed, unlike to T lymphocytes, the macrophages are more resistant to the cytopathic effects of HIV, and therefore macrophages are able to sustain the virus production for a longer period, thus significantly contributing to the spread of the virus in the host organism and its transmission between hosts [70]. This aspect makes the model predictions biologically relevant since the predicted oscillations period (up to 10 days) of the HIV molecular compoments cannot be realized in the cells with a shorter lifetime.

The developed mathematical model predicts the existence of oscillatory dynamics in HIV replication at a single cell level. To observe a similar dynamics of HIV growth in cell culture in vitro, a set of experiments specifically designed to observe the phenomenon is required using either synchronized cell populations or individual cells. Indeed, a population averaged measurements may smooth out and lose the appearance of oscillations. Obviously, these types of studies have not been done yet. However the predicted dynamics is consistent with the oscillatory patterns of HIV infection exhibited in the models describing the HIV infection of cell populations [71] and in tissue cultures [72]. In addition, the last study predicted the period of oscillations very close to the one observed in our model. We note that complex patterns of oscillatory behavior in HIV viremia and CD4+ T Cell Counts have been observed in HIV infected patients with the between peak intervals of about 20 days as reported in [73].

We propose that the identified oscillatory dynamics of HIV-1 replication, which is determined by the specific characteristics of the structural and functional organization of the viral genome, can be one of the possible mechanisms for the maintenance of the prolonged intracellular virus persistence. Indeed, let us consider the oscillatory behavior of the viral components from the viewpoint of a biological advantage. In our discussion we will follow a broadly accepted paradigm stating that if a specific feature of a biological system stably persists through many generations then this feature provides a certain evolutionary advantage in the struggle for survival. Now, the question is what type of benefit does the cycling dynamics of the viral components production would give to the virus as compared to a steady state one? In our view, the real advantage is that the likelihood of the survival of the HIV-1 infected cell population should increase.

Consider the population of infected macrophages bearing the provirus in a latent form. Assume that the cell population gets activated at some time. The activation will lead to the synthesis of the viral RNAs and proteins, the assembly and budding of the virus particles in every infected cell. Such a cell becomes a target for the immune system with the likelihood of the cell to be recognized by the cytotoxic T lymphocytes (CTLs) being proportional to the concentration of the virus-specific antigens that are expressed on the cell surface by the major histocompatibility complex (MHC) class I molecules. It is clear that in the absence of specific hiding mechanisms, the probability of the infected cell to be recognized and destroyed will be larger for a higher replication rate of the virus in the cell. Therefore, after some time most of the infected cells would become highly prone to killing by the immune system and this should lead to a complete elimination of the infected cell population.

Now assume that the HIV-1 life cycle follows an oscillatory behavior. Then the level of the virus production by every infected cell will cycle, and in the absence of the replication cycles synchronization between the cells these oscillations in the infected cell population will take place asynchronously. This implies that at any given time only a fraction of infected cells will be actively producing virus, with the rest of them staying in a silent mode of the virus replication. Obviously, the cells characterized by a low level virus replication will be less recognizable for the immune system as compared to the actively producing ones. Thus the immune system will recognize/destroy only a fraction of infected cells at any given time. Therefore, the oscillatory dynamics of the viral ontogenesis should increase the survival of the virus under the selection pressure by immune system.

Additional mechanism contributing to the long-term viral persistence in an infected cell may be linked to the oscillations in the level of the viral regulatory protein Nef. Indeed, it has been shown that Nef induces a reduction of the MHC class I molecules at the cell surface via endocytosis [74,75]. This in turn reduces the efficacy of the recognition and killing of the infected cells by CTLs leading to a latent infection characterized by a long term low level viral replication.

It should be noted that an advantageous impact of the oscillatory phenotype of the viral ontogeny on its evolutionary robustness goes beyond the above arguments. There is another phenomenon which we call the 'recovery effect'. Indeed, an active viral production by the infected cell over a long period of time can lead to the exhaustion of its resources and finally, to death. Therefore, the alternating phases of high- and low level virus replication should allow the cell to recover the consumed resources and to circumvent the death. Overall, this phenotype should increase the survival of the virus and it is evolutionary advantageous.

The oscillatory dynamics is an inherent feature of biological systems being a subject of mathematical analysis and prediction since the early modelling work on Lotka-Volterra. The mechanisms underlying the emergence of oscillations and their implications should be explored specifically for a given biological system. Oscillations are considered as an emergent property of adaptive biological systems. Following the general discussion of the potential advantages of oscillations for evolutionary selection and stabilization as presented recently in [76], one can propose that as far as the HIV infection is concerned, the oscillatory behavior of HIV replication can lead to an increase of the robustness of the virus persistence in the face of the protective immune responses.

The study is based on the mathematical model formulated with ODEs assuming that the changes of the reactants are continuous and deterministic. However, at low numbers of the molecules the consideration of the randomness and discreteness of the interactions needs to be taken into account. The application of stochastic modelling approaches to the Tat-Rev regulatory circuit of HIV-1 replication deserves a separate investigation.

In addition, we would like to note that the Tat-Rev regulatory system of HIV replication machinery contains all the structural motifs necessary for the emergence of the chaotic dynamics of the synthesis of viral components, including: the regulatory circuit of HIV-1 replication based on two feedback loops, one loop implementing a positive feedback regulation thus acting as an activator and the second one - a negative feedback regulation functioning as a repressor and a time lag in the synthesis of the regulatory factors, resulting from the fundamental processes of transcription, splicing, transport and translation [77-81]. Although in the analyzed domain of the model parameters the modelled system did not demonstrate chaotic regimes, we believe that the issue of an irregular behavior is very challenging and deserves further investigation.

The modelling results are obtained under a simplifying assumption that the immune system does not affect directly the infected cells. However, the interaction between the infected cells and the other cells of the immune system is a fundamental question. We plan to address this complex issue in future study.

## Conclusions

The developed mathematical model of HIV-1 replication predicts an oscillatory dynamics of the viral components synthesis in an infected cells for a broad range of the model parameters that results from the specific features of the structural-functional organization of the viral genome. The major processes underlying the emergence of the oscillations are the Rev protein re-cyclization at the nuclear pore complex, described by [35,36], and the robustness of the Rev-mRNA complex [59]. The biological advantage of the oscillatory dynamics in the intracellular synthesis of the viral components can be linked to the general evolutionary trend of developing mechanisms enabling the survival of a fraction of the infected target cells under the permanent pressure by the host immune system. This type of the ontogenesis dynamics, independently of other specific protection mechanisms evolved by the virus, can contribute to the long-term persistent production of the virus in the human organism, which is a remarkable property of HIV-1 [69].

## List of abbreviations used

HIV-1: human immunodeficiency virus type 1; TAR: transactivation responsive element;

RRE: rev response element.

## Competing interests

The authors declare that they have no competing interest.

## Authors' contributions

GAB and VAC conceived and managed the study. VAL, SIB and TMK developed the model and analyzed the data. VAL, TMK and SIB evaluated the model parameters. VAL and IAG performed model calculations. TMK and VAL wrote the manuscript with editing by SIB, IAG, GAB and VAC. All authors read and approved the final manuscript.

## Declarations

Publication of this article has been funded by the Programme of the Russian Academy of Sciences "Basic research for Medicine".

This article has been published as part of *BMC Genomics *Volume 15 Supplement 12, 2014: Selected articles from the IX International Conference on the Bioinformatics of Genome Regulation and Structure\Systems Biology (BGRS\SB-2014): Genomics. The full contents of the supplement are available online at http://www.biomedcentral.com/bmcgenomics/supplements/15/S12.

## References

[B1] Nisbet RM Gurney WSC Modelling fluctuating populations 1982 John Wiley & Sons: Chicesher

[B2] Keener J Sneyd J Mathematical Physiology 1998 Springer Verlag: New York

[B3] Voit EV Computational Analysis of Biochemical Systems 2000 Cambridge University Press

[B4] Fall CP Marland ES Wagner JM Tyson JJ Computational Cell Biology 2002 Springer Verlag: New York

[B5] Klevecz RR Li CM Marcus I Frankel PH Collective behavior in gene regulation: the cell is an oscillator, the cell cycle a developmental process FEBS J 2008 275 10 2372 2384 10.1111/j.1742-4658.2008.06399.x 18410382 PMC2858570

[B6] Leite MC Wang Y Multistability, oscillations and bifurcations in feedback loops Math Biosci Eng 2010 7 1 83 97 20104950 10.3934/mbe.2010.7.83

[B7] Hoffmann A Levchenko A Scott ML Baltimore D The IkappaB-NF-kappaB signaling module: temporal control and selective gene activation Science 2002 298 1241 1245 10.1126/science.1071914 12424381

[B8] Nelson DE Ihekwaba AEC Elliott M Johnson JR Gibney CA Foreman BE Nelson G See V Horton CA Spiller DG Edwards SW McDowell HP Unitt JF Sullivan E Grimley R Benson N Broomhead D Kell DB White MR Oscillations in NF-kappaB signaling control the dynamics of gene expression Science 2004 306 704 708 10.1126/science.1099962 15499023

[B9] Wang Y Paszek P Horton CA Kell DB White MR Broomhead DS Muldoon MR Interactions among oscillatory pathways in NF-kappa B signaling BMC Syst Biol 2011 5 23 10.1186/1752-0509-5-23 21291535 PMC3050740

[B10] Hirata H Yoshiura S Ohtsuka T Bessho Y Harada T Yoshikawa K Kageyama R Oscillatory expression of the bHLH factor Hes1 regulated by a negative feedback loop Science 2002 298 840 843 10.1126/science.1074560 12399594

[B11] Kageyama R Yoshiura S Masamizu Y Niwa Y Ultradian oscillators in somite segmentation and other biological events Cold Spring Harb Symp Quant Biol 2007 72 451 457 10.1101/sqb.2007.72.012 18419304

[B12] Bose I Ghosh B The p53-MDM2 network: from oscillations to apoptosis J Biosci 2007 32 5 991 997 17914240 10.1007/s12038-007-0103-3

[B13] Leloup JC Goldbeter A Toward a detailed computational model for the mammalian circadian clock Proc Natl Acad Sci USA 2003 100 7051 7056 10.1073/pnas.1132112100 12775757 PMC165828

[B14] Hayot F Jayaprakash C NF-kappaB oscillations and cell-to-cell variability J Theor Biol 2006 240 4 583 591 10.1016/j.jtbi.2005.10.018 16337239

[B15] Krishna S Jensen MH Sneppen K Minimal model of spiky oscillations in NF-kappaB signaling Proc Natl Acad Sci USA 2006 103 10840 10845 10.1073/pnas.0604085103 16829571 PMC1544135

[B16] Pigolotti S1 Krishna S Jensen MH Oscillation patterns in negative feedback loops Proc Natl Acad Sci USA 2007 104 16 6533 6537 10.1073/pnas.0610759104 17412833 PMC1871820

[B17] Jensen PB1 Pedersen L Krishna S Jensen MH A Wnt oscillator model for somitogenesis Biophys J 2010 98 6 943 950 10.1016/j.bpj.2009.11.039 20303851 PMC2849083

[B18] Gyuris A Vajda G Földes I Establishment of an MT4 cell line persistently producing infective HIV-1 particles Acta Microbiol Hung 1992 39 271 279 1364232

[B19] Karn J Stoltzfus CM Transcriptional and posttranscriptional regulation of HIV-1 gene expression Cold Spring Harb Perspect Med 2012 2 2 a006916 22355797 10.1101/cshperspect.a006916 PMC3281586

[B20] Laspia MF Rice AP Mathews MB HIV-1 Tat protein increases transcriptional initiation and stabilizes elongation Cell 1989 59 2 283 292 10.1016/0092-8674(89)90290-0 2553266

[B21] Marciniak RA Calnan BJ Frankel AD Sharp PA HIV-1 Tat protein trans-activates transcription *in vitro* Cell 1990 63 4 791 802 10.1016/0092-8674(90)90145-5 2225077

[B22] Feinberg MB Baltimore D Frankel AD The role of Tat in the human immunodeficiency virus life cycle indicates a primary effect on transcriptional elongation Proc Natl Acad Sci USA 1991 88 9 4045 4049 10.1073/pnas.88.9.4045 2023953 PMC51590

[B23] Harrich D Hsu C Race E Gaynor RB Differential growth kinetics are exhibited by human immunodeficiency virus type 1 TAR mutants J Virol 1994 68 9 5899 5910 8057469 10.1128/jvi.68.9.5899-5910.1994 PMC236995

[B24] Chojnacki J Müller B Investigation of HIV-1 assembly and release using modern fluorescence imaging techniques Traffic 2013 14 1 15 24 22957540 10.1111/tra.12006

[B25] Purcell DF Martin MA Alternative splicing of human immunodeficiency virus type 1 mRNA modulates viral protein expression, replication, and infectivity J Virol 1993 67 11 6365 6378 8411338 10.1128/jvi.67.11.6365-6378.1993 PMC238071

[B26] Richard N Iacampo S Cochrane A HIV-1 Rev is capable of shuttling between the nucleus and cytoplasm Virology 1994 204 1 123 131 10.1006/viro.1994.1516 8091647

[B27] Love DC Sweitzer TD Hanover JA Reconstitution of HIV-1 rev nuclear export: independent requirements for nuclear import and export Proc Natl Acad Sci USA 1998 95 18 10608 10613 10.1073/pnas.95.18.10608 9724751 PMC27942

[B28] Pond SJ Ridgeway WK Robertson R Wang J Millar DP HIV-1 Rev protein assembles on viral RNA one molecule at a time Proc Natl Acad Sci USA 2009 106 5 1404 1408 10.1073/pnas.0807388106 19164515 PMC2635779

[B29] Felber BK Drysdale CM Pavlakis GN Feedback regulation of human immunodeficiency virus type 1 expression by the Rev protein. J Virol 1990 64 3734 3741 2196381 10.1128/jvi.64.8.3734-3741.1990 PMC249668

[B30] Hammond BJ Quantitative study of the control of HIV-1 gene expression J Theor Biol 1993 163 2 199 221 10.1006/jtbi.1993.1117 8246504

[B31] Reddy B Yin J Quantitative intracellular kinetics of HIV Type 1 AIDS Res Hum Retroviruses 1999 15 3 273 283 10.1089/088922299311457 10052758

[B32] Kim H Yin J Robust growth of human immunodeficiency virus type 1 (HIV-1) Biophys J 2005 89 4 2210 2221 10.1529/biophysj.104.058438 16055539 PMC1366724

[B33] Tameru B Habtemariam T Nganwa D Ayanwale L Beyene G Robnett V Wilson W Computational modelling of intracellular viral kinetics and CD4+ cellular population dynamics of HIV/AIDS Adv Syst Sci Appl 2008 8 1 40 45 20448836 PMC2864528

[B34] Zarrabi N Mancini E Tay J-C Shahand S Sloot PMA Modeling HIV-1 intracellular replication: two simulation Procedia Computer Science 2012 1 1 555 564

[B35] Hutten S Kehlenbach RH Nup214 is required for CRM1-dependent nuclear protein export *in vivo* Mol Cell Biol 2006 26 18 6772 6785 10.1128/MCB.00342-06 16943420 PMC1592874

[B36] Hutten S Wälde S Spillner C Hauber J Kehlenbach RH The nuclear pore component Nup358 promotes transportin-dependent nuclear import J Cell Sci 2009 122 1100 1110 10.1242/jcs.040154 19299463

[B37] Gear CW Automatic integration of ordinary differential equations Communications of the ACM 1971 14 3 176 179 10.1145/362566.362571

[B38] Singh J Padgett RA Rates of *in situ *transcription and splicing in large human genes Nat Struct Mol Biol 2009 16 11 1128 1133 10.1038/nsmb.1666 19820712 PMC2783620

[B39] Wada Y Ohta Y Xu M Tsutsumi S Minami T Inoue K Komura D Kitakami J Oshida N Papantonis A Izumi A Kobayashi M Meguro H Kanki Y Mimura I Yamamoto K Mataki C Hamakubo T Shirahige K Aburatani H Kimura H Kodama T Cook PR Ihara S A wave of nascent transcription on activated human genes Proc Natl Acad Sci USA 2009 106 18357 18361 10.1073/pnas.0902573106 19826084 PMC2761237

[B40] Veloso A Kirkconnell KS Magnuson B Biewen B Paulsen MT Wilson TE Ljungman M Rate of elongation by RNA polymerase II is associated with specific gene features and epigenetic modifications Genome Res 2014 24 6 896 905 10.1101/gr.171405.113 24714810 PMC4032854

[B41] Boireau S Maiuri P Basyuk E de la Mata M Knezevich A Pradet-Balade B Backer V Kornblihtt A Marcello A Bertrand E The transcriptional cycle of HIV-1 in real-time and live cells J Cell Biol 2007 179 291 304 10.1083/jcb.200706018 17954611 PMC2064765

[B42] Maiuri P Knezevich A Bertrand E Marcello A Real-time imaging of the HIV-1 transcription cycle in single living cells Methods 2011 53 1 62 67 10.1016/j.ymeth.2010.06.015 20600934

[B43] Bohan CA Kashanchi F Ensoli B Buonaguro L Boris-Lawrie KA Brady JN Analysis of Tat transactivation of human immunodeficiency virus transcription *in vitro* Gene Expr 1992 2 4 391 407 1282057 PMC6057369

[B44] Graeble MA Churcher MJ Lowe AD Gait MJ Karn J Human immunodeficiency virus type 1 transactivator protein, tat, stimulates transcriptional read-through of distal terminator sequences *in vitro* Proc Natl Acad Sci USA 1993 90 13 6184 6188 10.1073/pnas.90.13.6184 8327498 PMC46892

[B45] Slice LW Codner E Antelman D Holly M Wegrzynski B Wang J Toome V Hsu MC Nalin CM Characterization of recombinant HIV-1 Tat and its interaction with TAR RNA Biochemistry 1992 31 48 12062 12068 10.1021/bi00163a014 1457403

[B46] Richter S Cao H Rana TM Specific HIV-1 TAR RNA loop sequence and functional groups are required for human cyclin T1-Tat-TAR ternary complex formation Biochemistry 2002 41 20 6391 6397 10.1021/bi0159579 12009901

[B47] Krombach F Münzing S Allmeling AM Gerlach JT Behr J Dörger M Cell size of alveolar macrophages: an interspecies comparison Environ Health Perspect 1997 105 Suppl 5 1261 1263 10.1289/ehp.97105s51261 9400735 PMC1470168

[B48] Audibert A Weil D Dautry F *In vivo *kinetics of mRNA splicing and transport in mammalian cells Mol Cell Biol 2002 22 6706 6718 10.1128/MCB.22.19.6706-6718.2002 12215528 PMC134034

[B49] Kessler O Jiang Y Chasin LA Order of intron removal during splicing of endogenous adenine phosphoribosyltransferase and dihydrofolate reductase premRNA Mol Cell Biol 1993 13 6211 6222 8413221 10.1128/mcb.13.10.6211-6222.1993 PMC364680

[B50] Allen TD Cronshaw JM Bagley S Kiseleva E Goldberg MW The nuclear pore complex: mediator of translocation between nucleus and cytoplasm J Cell Sci 2000 113 1651 1659 10769196 10.1242/jcs.113.10.1651

[B51] Grünwald D Singer RH *In vivo *imaging of labelled endogenous β-actin mRNA during nucleocytoplasmic transport Nature 2010 467 7315 604 607 10.1038/nature09438 20844488 PMC3005609

[B52] Mann DA Mikaélian I Zemmel RW Green SM Lowe AD Kimura T Singh M Butler PJ Gait MJ Karn J A molecular rheostat. Co-operative rev binding to stem I of the rev-response element modulates human immunodeficiency virus type-1 late gene expression J Mol Biol 1994 241 2 193 207 10.1006/jmbi.1994.1488 8057359

[B53] Daugherty MD Booth DS Jayaraman B Cheng Y Frankel AD HIV Rev response element (RRE) directs assembly of the Rev homooligomer into discrete asymmetric complexes Proc Natl Acad Sci USA 2010 107 28 12481 12486 10.1073/pnas.1007022107 20616058 PMC2906596

[B54] Efthymiadis A Briggs LJ Jans DA The HIV-1 Tat nuclear localization sequence confers novel nuclear import properties J Biol Chem 1998 273 3 1623 1628 10.1074/jbc.273.3.1623 9430704

[B55] Arava Y Wang Y Storey JD Liu CL Brown PO Herschlag D Genome-wide analysis of mRNA translation profiles in Saccharomyces cerevisiae Proc Natl Acad Sci USA 2003 100 7 3889 3894 10.1073/pnas.0635171100 12660367 PMC153018

[B56] MacKay VL Li X Flory MR Turcott E Law GL Serikawa KA Xu XL Lee H Goodlett DR Aebersold R Zhao LP Morris DR Gene expression analyzed by high-resolution state array analysis and quantitative proteomics: response of yeast to mating pheromone Mol Cell Proteomics 2004 3 5 478 489 10.1074/mcp.M300129-MCP200 14766929

[B57] Malim MH Cullen BR Rev and the fate of pre-mRNA in the nucleus: implications for the regulation of RNA processing in eukaryotes Mol Cell Biol 1993 13 6180 6189 8105371 10.1128/mcb.13.10.6180-6189.1993 PMC364677

[B58] Felber BK Hadzopoulou-Cladaras M Cladaras C Copeland T Pavlakis GN Rev protein of human immunodeficiency virus type 1 affects the stability and transport of the viral mRNA Proc Natl Acad Sci USA 1989 86 1495 1499 10.1073/pnas.86.5.1495 2784208 PMC286723

[B59] Dowling D Nasr-Esfahani S Tan CH O'Brien K Howard JL Jans DA Purcell DF Stoltzfus CM Sonza S HIV-1 infection induces changes in expression of cellular splicing factors that regulate alternative viral splicing and virus production in macrophages Retrovirology 2008 5 18 10.1186/1742-4690-5-18 18241354 PMC2267807

[B60] Kubota S Duan L Furuta RA Hatanaka M Pomerantz RJ Nuclear preservation and cytoplasmic degradation of human immunodeficiency virus type 1 Rev protein J Virol 1996 70 1282 1287 8551596 10.1128/jvi.70.2.1282-1287.1996 PMC189944

[B61] Kjems J Frankel AD Sharp PA Specific regulation of mRNA splicing *in vitro *by a peptide from HIV-1 Rev Cell 1991 67 1 169 178 10.1016/0092-8674(91)90580-R 1913815

[B62] Kjems J Sharp PA The basic domain of Rev from human immunodeficiency virus type 1 specifically blocks the entry of U4/U6.U5 small nuclear ribonucleoprotein in spliceosome assembly J Virol 1993 67 8 4769 4776 8331728 10.1128/jvi.67.8.4769-4776.1993 PMC237863

[B63] Pollard VW Malim MH The HIV-1 Rev protein Annu Rev Microbiol 1998 52 491 532 10.1146/annurev.micro.52.1.491 9891806

[B64] Carlotti F Dower SK Qwarnstrom EE Dynamic shuttling of nuclear factor kappa B between the nucleus and cytoplasm as a consequence of inhibitor dissociation J Biol Chem 2000 275 52 41028 41034 10.1074/jbc.M006179200 11024020

[B65] Lipniacki T Paszek P Brasier AR Luxon B Kimmel M Mathematical model of NF-kappaB regulatory module J Theor Biol 2004 228 2 195 215 10.1016/j.jtbi.2004.01.001 15094015

[B66] Jung A Maier R Vartanian JP Bocharov G Jung V Fischer U Meese E Wain-Hobson S Meyerhans A Recombination: Multiply infected spleen cells in HIV patients Nature 2002 418 6894 144 10.1038/418144a 12110879

[B67] Fernandes J Jayaraman B Frankel A The HIV-1 Rev response element: an RNA scaffold that directs the cooperative assembly of a homo-oligomeric ribonucleoprotein complex RNA Biol 2012 9 1 6 11 10.4161/rna.9.1.18178 22258145 PMC3342944

[B68] Daugherty MD D'Orso I Frankel AD A solution to limited genomic capacity: Using adaptable binding surfaces to assemble the functional HIV Rev oligomer on RNA Mol Cell 2008 31 824 834 10.1016/j.molcel.2008.07.016 18922466 PMC2651398

[B69] Klatt NR Chomont N Douek DC Deeks SG Immune activation and HIV persistence: Implications for curative approaches to HIV infection Immunol Rev 2013 254 1 326 342 10.1111/imr.12065 23772629 PMC3694608

[B70] Carter CA Ehrlich LS Cell biology of HIV-1 infection of macrophages Annu Rev Microbiol 2008 62 425 443 10.1146/annurev.micro.62.081307.162758 18785842

[B71] Nelson GW Perelson AS Modeling defective interfering virus therapy for AIDS: conditions for DIV survival Math Biosci 1995 125 2 127 153 10.1016/0025-5564(94)00021-Q 7881191

[B72] Spouge JI Shrager RI Dimitrov DS HIV-1 infection kinetics in tissue cultures Math Biosci 1996 138 1 1 22 10.1016/S0025-5564(96)00064-8 8942173

[B73] Kitchen CMR Yeghiazarian L Hoh R McCune JM Sinclair E Martin JN Deeks SG Immune activation, CD4+ T cell counts, and viremia exhibit oscillatory patterns over time in patients with highly resistant HIV infection PLoS One 2011 6 6 e21190 10.1371/journal.pone.0021190 21701594 PMC3118814

[B74] Schaefer MR Wonderlich ER Roeth JF Leonard JA Collins KL HIV-1 Nef Targets MHC-I and CD4 for Degradation Via a Final Common β-COP-Dependent Pathway in T Cells PLoS Pathog 2008 4 8 e1000131 10.1371/journal.ppat.1000131 18725938 PMC2515349

[B75] Schwartz O Marechal V Le Gall S Lemonnier F Heard JM Endocytosis of major histocompatibility complex class I molecules is induced by the HIV-1 Nef protein Nat Med 1996 2 3 338 342 10.1038/nm0396-338 8612235

[B76] Cheong R1 Levchenko A Oscillatory signaling processes: the how, the why and the where Curr Opin Genet Dev 2010 20 6 665 669 10.1016/j.gde.2010.08.007 20971631 PMC3895451

[B77] Goldbeter A Gonze D Houart G Leloup JC Halloy J Dupont G From simple to complex oscillatory behavior in metabolic and genetic control networks Chaos 2001 11 247 260 10.1063/1.1345727 12779458

[B78] Likhoshvai VA Fadeev SI Kogai VV Khlebodarova TM On the chaos in gene networks J Bioinform Comput Biol 2013 11 1 1340009 10.1142/S021972001340009X 23427991

[B79] Mackey MC Glass L Oscillation and chaos in physiological control systems Science 1977 197 287 289 10.1126/science.267326 267326

[B80] Martinez de la Fuente I Martinez L Veguillas J Aguirregabiria JM Quasiperiodicity route to chaos in a biochemical system Biophys J 1996 71 5 2375 2379 10.1016/S0006-3495(96)79431-6 8913578 PMC1233727

[B81] Zhang Z Ye W Qian Y Zheng Z Huang X Hu G Chaotic motifs in gene regulatory networks PLoS One 2012 7 7 e39355 10.1371/journal.pone.0039355 22792171 PMC3391214

[B82] Malim MH Cullen BR HIV-1 structural gene expression requires the binding of multiple Rev monomers to the viral RRE: implications for HIV-1 latency Cell 1991 65 2 241 248 10.1016/0092-8674(91)90158-U 2015625

[B83] Robert-Guroff M Popovic M Gartner S Markham P Gallo RC Reitz MS Structure and expression of tat-, rev-, and nef-specific transcripts of human immunodeficiency virus type 1 in infected lymphocytes and macrophages J Virol 1990 64 7 3391 3398 2191150 10.1128/jvi.64.7.3391-3398.1990 PMC249590

